# Agulhas Current properties shape microbial community diversity and potential functionality

**DOI:** 10.1038/s41598-018-28939-0

**Published:** 2018-07-12

**Authors:** Sandra Phoma, Surendra Vikram, Janet K. Jansson, Isabelle J. Ansorge, Don A. Cowan, Yves Van de Peer, Thulani P. Makhalanyane

**Affiliations:** 1 Centre for Microbial Ecology and Genomics (CMEG), Department of Biochemistry, Genetics and Microbiology, Natural Sciences 2, University of Pretoria, Pretoria, 0028 South Africa; 2 Earth and Biological Sciences Directorate, Pacific Northwest National Laboratories, P.O. Box 999, Richland, WA USA; 3 Department of Oceanography and Marine Research Institute (Ma-Re), University of Cape Town, Rondebosch, 7701 South Africa; 4 VIB Centre for Plant Systems Biology, B-9052 Ghent, Belgium; 5 Department of Plant Biotechnology and Bioinformatics, Ghent University, B-9052 Ghent, Belgium

## Abstract

Understanding the impact of oceanographic features on marine microbial ecosystems remains a major ecological endeavour. Here we assess microbial diversity, community structure and functional capacity along the Agulhas Current system and the Subtropical Front in the South Indian Ocean (SIO). Samples collected from the epipelagic, oxygen minimum and bathypelagic zones were analysed by 16S rRNA gene amplicon and metagenomic sequencing. In contrast to previous studies, we found high taxonomic richness in surface and deep water samples, but generally low richness for OMZ communities. Beta-diversity analysis revealed significant dissimilarity between the three water depths. Most microbial communities were dominated by marine Gammaproteobacteria, with strikingly low levels of picocyanobacteria. Community composition was strongly influenced by specific environmental factors including depth, salinity, and the availability of both oxygen and light. Carbon, nitrogen and sulfur cycling capacity in the SIO was linked to several autotrophic and copiotrophic Alphaproteobacteria and Gammaproteobacteria. Taken together, our data suggest that the environmental conditions in the Agulhas Current system, particularly depth-related parameters, substantially influence microbial community structure. In addition, the capacity for biogeochemical cycling of nitrogen and sulfur is linked primarily to the dominant Gammaproteobacteria taxa, whereas ecologically rare taxa drive carbon cycling.

## Introduction

Disentangling the influence of oceanographic factors, which shape marine microbial ecosystems, remains a major ecological endeavour. Perhaps unsurprisingly, little is known regarding the impacts of ocean currents (or frontal systems) on microbial communities and the functions they mediate. Early estimates suggest that oceans may harbour substantial levels of microbial cells: values of over 10^5^ per millimetre of seawater in the euphotic zone and extrapolated to over 10^28^ cells for global oceanic waters^1^. However, despite the numerical abundance of microbial cells, microbial communities remain relatively underexplored in some oceans due to logistical constraints associated with marine research^2,3^. Moreover, the complexity of the three-dimensional ocean ecosystem further complicates efforts to understand the impact of oceanographic features on marine microbiomes. For instance, a combination of water masses at varying depths, photic and aphotic zones, in addition to oceanic current and frontal systems, complicate attempts to understand how environmental factors influence the structures and ecosystem services of marine microbial communities^4^. Previous studies suggest that the diversity and composition of marine ecosystems may vary markedly with either geographic distance^5,6^, depth^7,8^, water mass endemicity^9–11^, environmental variables^12,13^, oceanic frontal zones^14^ or advection by ocean currents^15–18^. Consequently, further phylogenetic and metagenomics surveys of understudied marine ecosystems may clarify biogeographic patterns, environmental determinants and functional cues which shape microbial communities throughout the water column.

**Figure 1:**
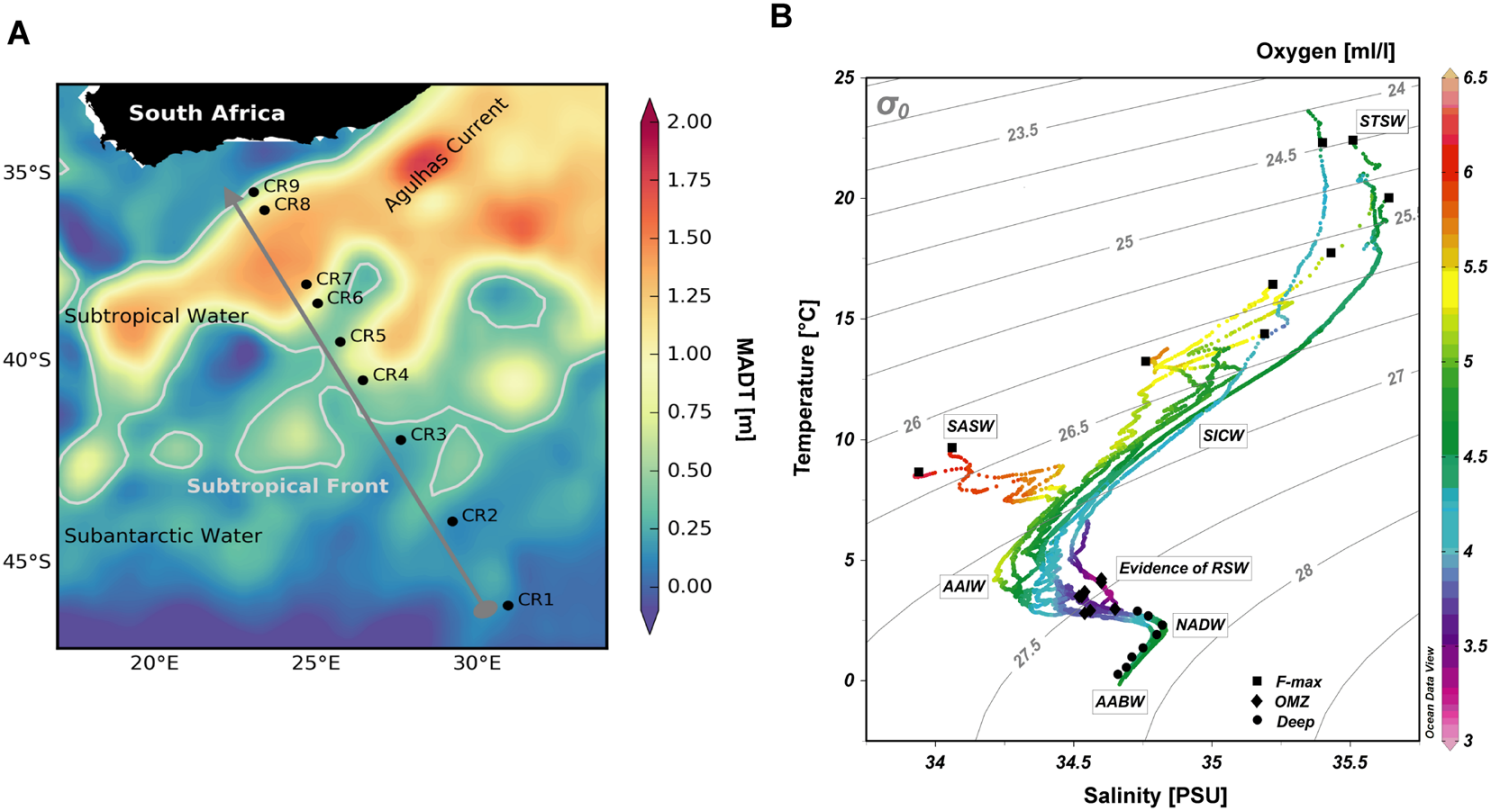
Agulhas Current circulation, location of Crossroads (CR) hydrographic stations and distribution of samples: (**A**) A map of the sampling area overlaid with the Crossroad cruise track (black line) and hydrographic stations (black bullets). The Agulhas Current system was determined using sea surface heights from Maps of Absolute Mean Dynamic Topography^95^ (MADT, in meters), with its position averaged over the duration of sampling (9–14 May 2015). The subtropical front indicated by grey lines^96^. Image courtesy of Marcel du Plessis. (**B**) An oceanographic profile of F-max, OMZ and Deep samples based on *in situ* measurements of temperature (°C), salinity (psu) and oxygen (m/l). **B** was generated using Ocean Data View (Schlitzer, R., Ocean Data View, http://odv.awi.de, 2017).

Western boundary currents are strong, narrow and wind-driven. They carry large volumes of warm subtropical waters poleward into the mid-latitudes^19,20^. These currents form a major component of global ocean circulation and climate variability. The Agulhas Current (AC) occurs within 20 km from the South African east coast, extending from 25°S to 40°S and forms a critical crossroad for inter-ocean exchange of heat, salt and biota between the Indian, Atlantic and Southern Oceans^19^. The AC has an estimated mean volume transport of 75 Sverdrups (Sv; one unit is equivalent to 1 × 10^6^ m^3^ of seawater per second), may extend to the upper 2300 m^21^, and exhibits flow velocities in excess of 2 ms^−1^ in surface waters^22–24^. At the southern tip of the continental shelf, the AC retroflects, flowing eastwards as the Agulhas Return Current (ARC)^25^ (Fig. 1A). Some leakage of the ARC-driven water between the South Indian and South Atlantic Oceans occurs through the generation of large warm, saline oligotrophic Agulhas rings, filaments and sub mesoscale processes^19,23,26,27^. These Agulhas rings transport low chlorophyll waters^28^ and significantly determine vertical mixing, which drives nitrogen cycling^29^. Agulhas mass continuity has also been shown to drive surface waters down, resulting in the upwelling of cold and nutrient waters south of the current. This in turn directly influences the metabolism and biogeochemical signatures^29^ and primary productivity^28^ of planktonic communities. While the impact of these currents on microbial communities remain unclear, it is reasonable to predict that the Agulhas Current system may influence microbial diversity and ecosystem functionality in the South Indian Ocean (SIO).

To elucidate microbial diversity and functional potential in the SIO, we undertook a metagenomic analysis of seawater samples collected along the Crossroads (CR) transect line^30^. This oceanographic monitoring line was established in 2013 to monitor air-sea exchange between the SIO and the South Atlantic Ocean. At each station, samples were collected from the epipelagic (fluorescence maximum, F-max), oxygen minimum zone (OMZ) and 10 m above the seafloor (Deep). Illumina (I-tag) sequencing of the 16S rRNA gene amplicons was used to assess the bacterial and archaeal community composition, structure and richness. Metagenomic sequencing was used to survey the microbial diversity and functional genes involved in carbon, nitrogen and sulfur cycling. Based on previous findings^29^, we propose that physicochemical variables across this current system may shape free-living picoplankton diversity and influence their potential metabolic capabilities.

## Results

### Oceanographic characteristics and site descriptions

Supplementary Tableses (n = 27, 9 × 3) were collected over a 6-day period to minimize seasonal fluctuations between sites. During the cruise, the subtropical Agulhas Current exhibited a high velocity of 2 ms^−1^ at approximately 36°S (station CR8). The Agulhas Return current meandered considerably during this cruise and was observed at CR6 (Fig. 1A) and again further south at ~39.5°S (station CR5), north of the Subtropical Front (STF) at 40°S^31,32^ along the CR line. The hydrographic stations selected as sampling points spanned key water masses in the SIO. A sharp increase in surface temperature and salinity (Supplementary Table S1) confirmed the change in oceanic zones as the South African research vessel Agulhas II (R/V S.A. Agulhas II) crossed the Subtropical Front between stations CR4 and CR5 (Fig. 1A). This frontal mixing zone separated the cool, fresher Subantarctic surface water (SASW) from the warmer subtropical surface water (STSW) located north of the STF. Consequently, these surface waters (F-max) showed gradually increasing *in situ* temperatures (from 8.66–22.31 °C), higher dissolved oxygen concentrations (from 4.47–6.10 ml/l) and a maximum *in situ* fluorescence of 2.7 mg/m^3^ (a proxy for chlorophyll representing phytoplankton biomass). Antarctic Intermediate water (AAIW), which form from the subduction of fresh Antarctic Surface water at the Polar Front, also demonstrated high levels of dissolved oxygen (>5.0 ml/l). However, CR stations within the Agulhas Current, displayed higher salinity (>34.50 psu) and oxygen minimum levels (average 3.73 ml/l) between the 800–1000 m range, providing evidence of Red Sea Water (RSW). RSW represent seawater which was advected southwards through the Mozambique Channel and carried within the southward fast flowing Agulhas Current. In the bathypelagic layers, Antarctic Bottom Water (Deep) showed an increase in salinity (average 34.75 psu) when compared to samples collected within the RSW (average of 34.55 psu). A map representation of the SIO samples, *in situ* temperature, and salinity against dissolved oxygen suggests the presence of distinct water masses (Fig. 1B).

### Taxonomic analysis of 16S rRNA gene amplicon sequences

**Figure 2:**
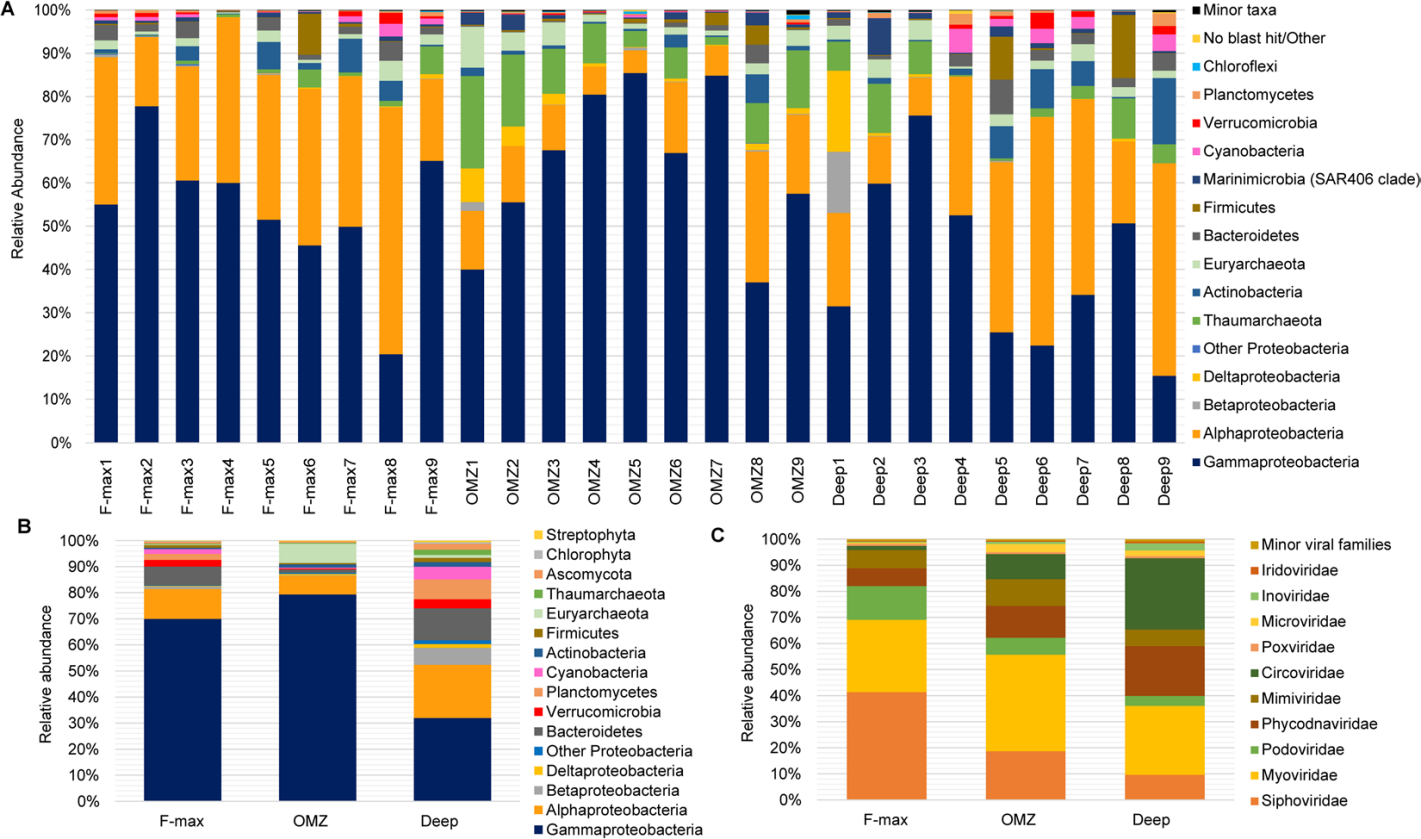
Taxonomic percent abundance of 16S rRNA gene amplicon and shotgun metagenomic reads (above 1%): (**A**) 16S rRNA gene, (**B**) Eukaryota, Archaea and Bacteria and (**C**) Viruses.

A total of 1,355,966 valid 16S rRNA gene sequences were generated (ranging from 34,397 to 65,324; standard deviation (SD) of ± 6,532). The rarefied 928,719 sequences (subsampled to 34,397, the minimum number of sequences in any sample) clustered into 2,925 (average of 649; SD of ± 155) bacterial and archaeal operational taxonomic units (OTUs) based on a 97% similarity cut-off (Supplementary Table S2). The relative abundances of bacterial lineages in the SIO was high (93.1% of the total OTUs assigned), with considerably lower proportions of Archaea (6.7%), and unassigned sequences (0.2%). Despite the comparatively high number of sequences per sample, rarefaction curves did not reach saturation, reflecting high phylogenetic diversity in these communities (Supplementary Fig. S1). Taxonomic analysis revealed a total of 21 phyla, which together constituted 88.7% of classified sequences. The relative abundances of bacteria was dominated by members of the phylum Proteobacteria (81.0%), followed by Actinobacteria (3.0%), Bacteroidetes (1.9%), Firmicutes (1.8%) and Cyanobacteria (1.0%), all of which appear to be ecologically rare (i.e., present at low abundances) (Fig. 2A). Gammaproteobacteria (mostly order Pseudomonadales), followed by Alphaproteobacteria (mostly SAR11 clade) were particularly common, being dominant in samples recovered from most stations and depths. Archaeal phyla were common across all samples, included Thaumarchaeota (5.7%; mostly Candidatus *Nitrosopelagicus*), followed by Euryarchaeota (2.8%; mostly class Marine group II (MGII)).

We observed a shift in dominant bacterial phyla within water masses along the CR transect. Overall, observable differences in bacterial taxonomy and relative abundances were found between the Agulhas Return Current (station CR5) and Agulhas Current (station CR8) for both F-max and OMZ samples (Fig. 2A). Gammaproteobacteria were dominant in SASW (Subantarctic zone surface water; 64%; stations CR1 and CR2), decreasing by 16% in STSW (Subtropical zone surface water; stations CR3-CR9). Members of class Gammaproteobacteria were also dominant in the OMZ (up to 85% of OTUs from the Agulhas Return Current and around 37% in Agulhas Current samples). Archaea (Thaumarchaeota and Euryarchaeota) made up to 31% of Subantarctic OMZ communities but were below 20% in subtropical OMZ waters. However, Deep communities differed markedly at each station.

The SIO core microbiome, here defined as individual OTUs which were present across all 27 CR samples, comprised of 14 OTUs (146, 395 sequence counts; 0.5% of all OTUs) representing a relative abundance of 15.8% of all sequences. The OTUs contributing to the core microbiome comprised of 8 Proteobacteria (4 Gammaproteobacteria and 4 Alphaproteobacteria classes) and 6 Archaea (3 Euryarchaeota and 3 Crenarchaeota phyla) (Supplementary Fig. S3).

### Microbial diversity and community structure

We assessed alpha diversity using OTU richness, Shannon, Simpson and Phylogenetic Diversity indices. Our analysis showed that alpha diversity was highest in Deep samples (Supplementary Table S2) and lowest in the OMZ associated with RSW. However, the OMZ and deep communities proximal to the Agulhas Current (CR8) displayed the highest alpha diversity values. In contrast, gamma diversity was higher for F-max than Deep and OMZ samples, at 8491, 8064 and 7444, respectively.

**Figure 3:**
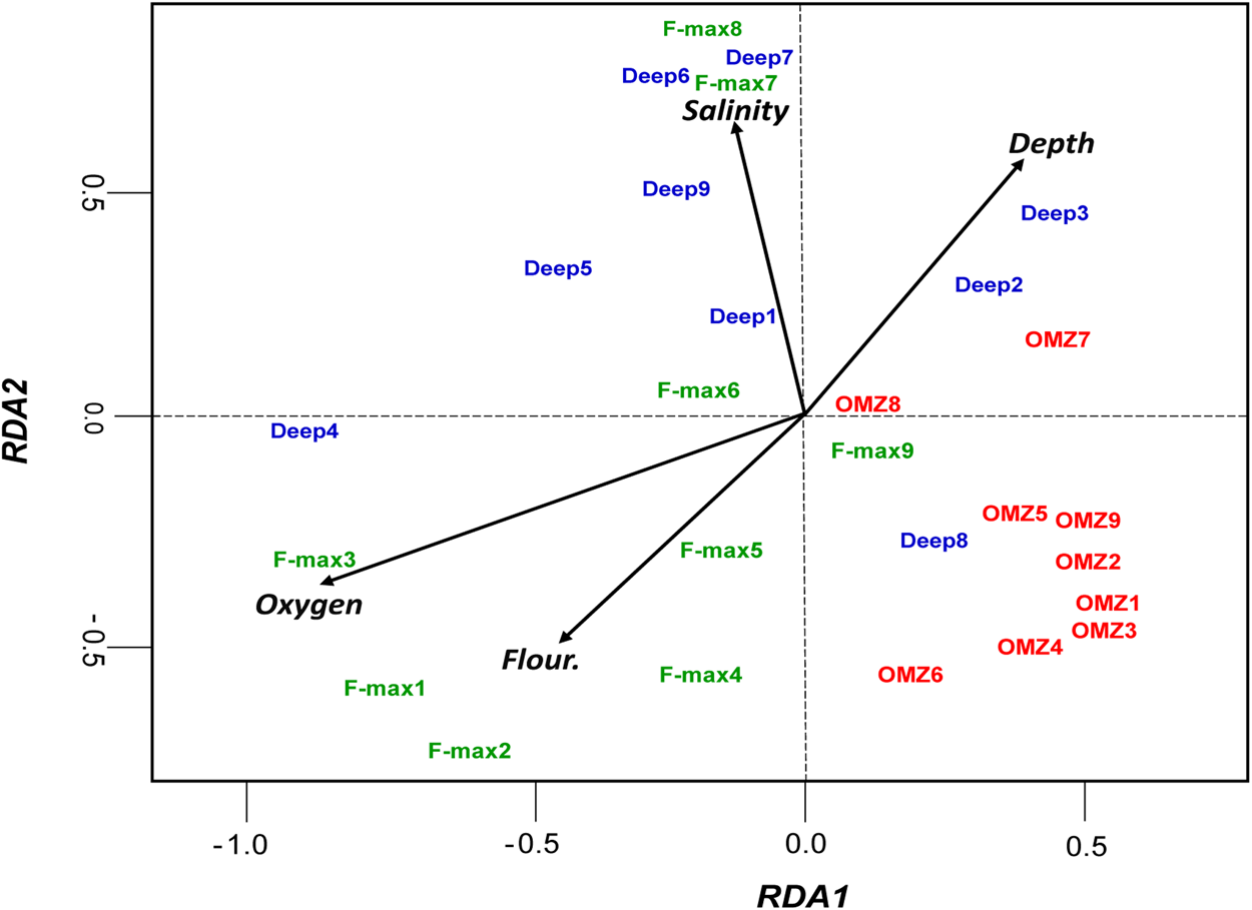
Relationship between microbial community composition and environmental parameters. RDA ordination described physicochemical variables (black arrows*, p* < 0.05) which significantly explained the variability in microbial community structure. The direction of the vectors indicates the effect of each driver variable on the two axes. The length of the arrow is proportional to the rate of change, whereas, the direction represents the correlations to the axes. Coloured text corresponds to sample depth: Deep, blue text; F-max, green and OMZ, red.

The NMDS ordination plot showed that F-max and Deep samples were broadly similar, compared to the OMZ communities (Supplementary Fig. S4). This observation was supported by two-way analysis of similarity (ANOSIM), which revealed that the F-max and Deep samples were not significantly different (ANOSIM R = 0.013; *p* > 0.05, adjusted using the Benjamini and Hochberg correction). However, the microbial communities in the OMZ samples varied substantially, in comparison to the F-max and Deep samples (Supplementary Table S3). We performed RDA to determine the effect of environmental variables on microbial community structure. We found that oxygen and fluorescence were the most important variables explaining the variability of the F-max communities, while depth appears to be an important factor structuring OMZ samples (Fig. 3). We used variation partitioning analysis to further explore the specific effects of environmental variables (salinity, oxygen and fluorescence) and depth on microbial community structure. Variation partitioning models showed that the environment alone explained 35% of the community variation. In contrast, the environment and depth collectively accounted for 32% of the community variation (Supplementary Fig. S5). Consistent with previous studies, a large proportion of variance was unexplained (65% and 68%, respectively).

**Figure 4:**
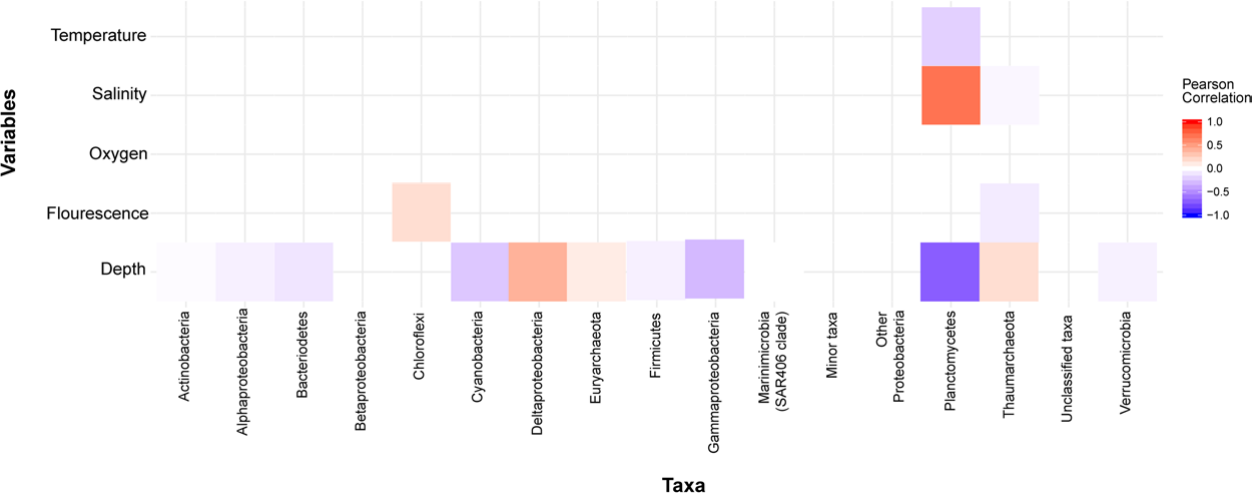
Pearson correlations between microbial communities and environmental variables shown as a heat map. A Pearson’s r value of 1 (red) indicates a total positive correlation, a value of -1 (blue) indicates a total negative correlation, and a value of 0 (white) indicates no correlation. *p* value adjusted to <0.05.

Pearson correlation coefficients (r; Supplementary Table S4) confirmed that several bacterial and archaeal phyla were significantly correlated with environmental variables including depth, fluorescence, salinity and temperature (Fig. 4). Cyanobacteria, Gammaproteobacteria and Planctomycetes were negatively correlated with depth, in contrast to Deltaproteobacteria and archaeal taxa, which were positively correlated (*p* < 0.05; *p* value corrected using Benjamini and Hochberg correction).

### Taxonomic analysis of metagenomic reads

To corroborate results from 16S rRNA gene analyses and to gain further insights into microbial biogeochemical capacity along depth profiles, we performed metagenome sequencing of three samples. DNA samples collected from each depth were pooled, irrespective of water masses and location, to test the effect of depth stratification in relation to functionality. Approximately 5.1, 4.8 and 4.6 Gbp of sequence data was generated for F-max, OMZ and Deep samples, respectively (Supplementary Table S5). The high quality merged reads (mean phred quality >20), obtained after removing ambiguous bases and exact duplicates, yielded 1.45, 0.68 and 0.85 Gbp of sequence data for the F-max, OMZ and Deep, respectively (Supplementary Table S6). Metaxa2 analysis of the resultant reads against the SILVA SSU reference database was broadly consistent with the 16S rRNA gene amplicon data, with a high relative abundance of the bacterial phyla (specifically Proteobacteria, Bacteroidetes, Firmicutes, and Actinobacteria) and low numbers of Archaea (specifically Euryarchaeota, Thaumarchaeota and Crenarchaeota). Consistent with the 16S rRNA gene amplicon data, the relative abundances of bacterial phyla across the three depths differed from each other (Supplementary Fig. S6). Functional annotation and taxonomic binning using MEGAN revealed a high relative abundance of Proteobacteria, Bacteroidetes, Firmicutes, Planctomycetes, Cyanobacteria and Actinobacteria with low relative abundances of other phyla (Fig. 2B). Euryarchaeota and Thaumarchaeota sequences were identified, albeit at very low relative abundances, especially in the OMZ and Deep samples. Reads were also assigned to members of the Eukarya, including Ascomycota, Chordata, Chlorophyta and Streptophyta (Fig. 3B). In addition, reads assigned to viral taxa were mostly affiliated to dsDNA viruses (i.e.; Myoviridae, Siphoviridae, Podoviridae and Phycodnaviridae). The OMZ metagenome harboured the lowest relative abundance of viral reads (Fig. 2C). The highest number of classified viral reads was from the F-max sample, followed by the Deep metagenome.

### Depth trends in biogeochemical cycling capacity

**Figure 5:**
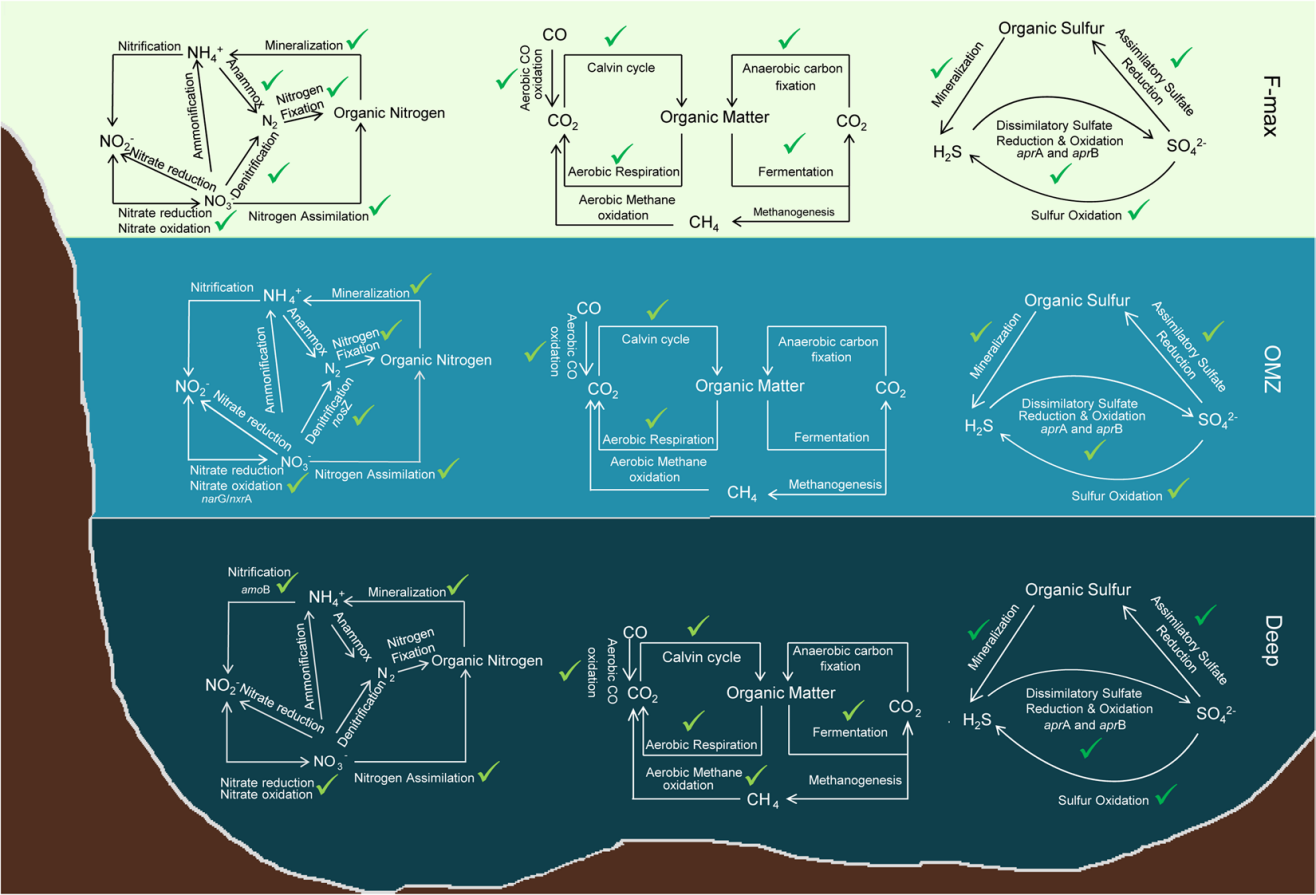
Functional breakdown of carbon, nitrogen and sulfur biogeochemical cycles present in each SO metagenome. Red crosses indicate the absence of specific pathways.

We analysed key marker genes involved in carbon, nitrogen and sulfur metabolism in the metagenomes. For biogeochemical pathway reconstruction, we selected key genes as previously described^33,34^, with some additional markers. Specifically, both Ribulose-1,5-bisphosphate carboxylase/oxygenase (RuBisCO) large and small subunits and phosphoribulokinase were selected as key marker genes for aerobic carbon fixation. The nitrogen metabolism pathways were examined using the Kyoto Encyclopedia of Genes and Genomes (KEGG) orthology identifiers for ammonification (*nrf*A), anammox (*hao*/*hzo*), denitrification (*nos*Z, *nor*B and *nor*C), nitrification (*amo*A, *amo*B and *amo*C), nitrogen fixation (*nif*D, *nif*K and *nif*H) and nitrate reduction (*nap*A and *nap*B) pathways. Genes linked to sulfur metabolism, including those for the assimilatory sulfate reduction (*cys*C, *cys*N and *cys*D), dissimilatory sulfate reduction (*apr*A, *apr*B and *dsr*A) and sulfide/thiosulfate (sox) pathways, were also found in all three metagenomes^34^ (Fig. 5).

### Carbon cycling capacity

Reads representing genes implicated in aerobic carbon fixation via the Calvin Cycle (e.g.; RuBisCO) were observed in samples from all depths. RuBisCO genes were similar to those previously identified^35^ in the bacterial taxa Cyanobacteria (*Synechococcus* sp. and *Prochlorococcus marinus*) and Proteobacteria (mostly *Rhodopseudomonas* sp.) (Supplementary Tables S7, S8 and S9). We found several sequences similar to those previously recovered from eukaryotic green algal Kingdom *Viridiplantae*, which suggested that members of this clade may contribute to aerobic carbon fixation in the F-max community (Supplementary Table S7). RuBisCO large subunit Open Reading Frames (ORFs) from metaSPAdes assembly were identified in the Deep (1 ORF; closest homology to *Synechococcus* sp.) and F-max (3 ORFs; *Rhodopseudomonas* sp., *Stichococcus* sp. and *Marivita hallyeonensisi*) metagenomes. Phylogenetic reconstructions showed that these sequences clustered with RuBisCO form I and IV proteins (Supplementary Fig. S7). Furthermore, a high number of photosystem I and II hits were observed in the F-max, compared to the OMZ and the Deep samples. Moreover, reads for photosystem I and II were mostly associated with the Cyanobacterial lineage, *Synechococcus*, with some genes assigned to *Viridiplantae*. A limited number of photosystem component reads were also assigned to marine viruses (Supplementary Table S10). Genes involved in anaerobic carbon fixation (via the Reductive Tricarboxylic Acid Cycle (rTCA)) were observed only in the F-max sample (Fig. 5). Methanogenesis and aerobic methane oxidation genes were not observed in any of the metagenomes. However, fermentative pathway enzymes were present in F-max and deep samples and were affiliated with Planctomycetaceae (Supplementary Tables S7 and S9). Most carbon monoxide (CO) oxidation pathway genes were present in all samples showing similarity to those previously reported from Actinobacteria, Planctomycetaceae and Proteobacteria (Alphaproteobacteria, Gammaproteobacteria and Betaproteobacteria) (Supplementary Tables S7, S8 and S9).

### Nitrogen cycling capacity

Nitrogen fixation genes from Proteobacteria (mostly Alphaproteobacteria (*Rhodopseudomonas* sp.)) were only found in F-max and OMZ samples (Supplementary Tables S7 and S8). Nitrification pathway genes were affiliated to unclassified Betaproteobacteria (Deep) and unclassified Thaumarchaeota (F-max), respectively (Supplementary Tables S9 and S7). Denitrification pathway genes were observed in F-max and OMZ metagenomes (Fig. 5). Organic nitrogen assimilation genes, mostly affiliated to bacteria and some archaea (Thaumarchaeota) and eukaryotic green algae (division Chlorophyta), were identified in all metagenomes. A combination of nitrate reduction and oxidation pathway genes (i.e.; *nar*G/*nxr*A and *nar*H/*nxr*B) were mostly associated to members of the class Gammaproteobacteria and unclassified Alphaproteobacteria in the F-max and deep metagenomes (Supplementary Table S7). Moreover, denitrification pathway genes were observed in F-max and OMZ samples (Fig. 5; Supplementary Table S7 and Supplementary Table S8). Nitrogen mineralization genes were mostly associated with the bacterial, archaeal and some eukaryotic lineages (i.e.; unknown Archaea, Proteobacteria, Planctomycetes, Verrucomicrobia, Actinobacteria, Bacteroidetes, and Viridiplantae) across all metagenomes.

### Sulfur cycling capacity

In comparison to the carbon and nitrogen cycles, sulfur metabolism pathways were complete (Fig. 5). Key genes linked to sulfur oxidation, dissimilatory sulfate reduction and oxidation were similar to those reported from Alphaproteobacteria (unclassified Pelagibacteraceae; Supplementary Tables S7, S9 and S9). Genes linked to potential mineralization of organic sulfur compounds were identified in all metagenomes. Polysulfide reductase genes (*psr*A) were affiliated to diverse bacterial lineages including Verrucomicrobia, the Terrabacteria group, Actinobacteria and unclassified bacteria, in both the F-max and Deep samples (Supplementary Tables S7 and S9). The F-max metagenome harboured the most reads for Sox pathway genes (i.e.; *sox*A, *sox*B, *sox*D, *sox*H, *sox*X, *sox*Y and *sox*Z), affiliated to members of Alphaproteobacteria, Gammaproteobacteria and, to a lesser extent, Betaproteobacteria (Supplementary Table S11).

## Discussion

The Agulhas Current system plays a pivotal role in regulating thermohaline circulation^26,36^. However, very little is known of how the system may impact marine microbiomes and the ecosystem services they provide. While a number of studies have analysed surface and sediment marine samples, very few have assessed the influence of water mass variability on microbial diversity and functional capacity^6,7,12–14,17,18,37–39^. None of these studies has assessed community dynamics in the understudied South Indian Ocean, which is dominated by the Agulhas Current system. By providing the first austral autumn analysis of South Indian Ocean microbial communities (using 16S rRNA gene amplicon sequencing) and their functional capacity (using metagenomic reads), we provide insights into microbial diversity and capacity for biogeochemical cycling in this important marine ecosystem. We demonstrate that free living picoplankton, dominated by chemoautotrophic taxa and were notably shaped by specific environmental variables in this current system.

### Taxonomic diversity dominated by Gammaproteobacteria

Proteobacteria was the dominant bacterial phylum identified in this study. Specifically, the F-max communities were dominated by Gammaproteobacteria, followed by Alphaproteobacteria. Gammaproteobacterial dominance in Indian Ocean surface water masses has also been reported by Gupta and colleagues^38–40^. This is in contrast to other reports, which have shown that marine surfaces are dominated by members of the SAR11 clade (class Alphaproteobacteria)^41^. Gammaproteobacteria appear to dominate in marine environments with high levels of dissolved organic matter (DOM), whereas SAR11 are prevalent in nutrient limited systems. In regions such as the SIO, it is reasonable to predict that SAR11 may be outcompeted as a result of high DOM turnover rates, which favour phytoplankton blooms^42^. This extrapolation is supported by previous studies in Australian Antarctic waters, which have also shown a generally weak inverse correlation between the relative abundance of SAR11 with chlorophyll-*a* fluorescence levels^43^.

A number of studies have also shown generally low relative abundance levels of Cyanobacteria in waters within the vicinity of the SO^44^. However, the precise reasons for the paucity of members of this phylum in these regions remain unclear^44^. We suggest that the environmental conditions in the SO may directly influence the relative abundance of photoautotrophic bacterial lineages such as cyanobacteria. The Indian sector of the SO (~30–50°S) has a low chlorophyll content, low levels of trace elements (e.g., nickel, zinc, copper and iron) and relatively higher nutrient levels^45,46^, in comparison to waters in other marine regions. These low micronutrient levels are likely to limit the development of cyanobacterial populations in surface waters^44,47^. The detection of cyanobacteria in Deep samples may be due to ‘legacy DNA’ resulting from the Agulhas Current advection. Similarly, previous studies have suggested that primary productivity and the associated dominance of eukaryotic phytoplankton along the Agulhas Current-driven SIO may be highly constrained^47,48^. There is some evidence to support this assertion, with several studies showing that increased micronutrient availability may directly lead to more efficient growth of cyanobacteria and phytoplankton^49^.

Excluding the highly abundant Gamma- and Alphaproteobacteria, both Deep and OMZ samples harboured several other taxa with known chemolithoautotrophic lifestyles; e.g., carbon fixing and sulfur-oxidizing *Oceanospirillales*, Thaumarchaeota and anammox Planctomycetes, as observed in previous studies^50^. Our findings support a recent metaproteomic based survey of sites across OMZ, which demonstrated a prevalence of anammox Planctomycetes, the distribution of which were also highly significantly correlated with depth^51^. We also demonstrate that other bacterial and archaeal phyla were significantly correlated with depth, which is consistent with previous studies^52^.

The viral community structure mirrored fluorescence profiles (i.e., highest diversity at F-max followed by Deep and OMZ, respectively). This suggests that the structure of viral communities was consistent with phototrophic and heterotrophic bacterial abundance, as reported previously^53,54^. Viruses (mostly dsDNA, affiliated to the order *Caudovirales*) harboured photosynthetic genes, suggesting their potential role in influencing genetic and functional diversity of bacterial lineages^55^. Viruses have been shown to increase prokaryotic diversity within marine habitats^56^ and are likely to influence the cycling of nitrogen and sulfur in the epipelagic zone^57^. Bacteriophages may also play an important role in the diversification of class Gammaproteobacteria^53,55^, which dominate the SIO marine water. While our metagenomic data provide insights into viral diversity in SIO marine waters, the precise mechanisms used by viral communities to influence microbial community structure in oligotrophic regions warrants further investigation.

### Vertical and horizontal trends in microbial community structure

Free-living microbial communities found in the Deep samples were more diverse than those recovered from the F-max and OMZ samples, which harboured lower levels of alpha diversity. This result is in broad agreement with a recent global survey of marine microbial communities^2^. However, several studies which have assessed microbial community composition across depth strata have yielded conflicting results^2,58–61^. This is likely to be due to the distinctive environmental conditions (e.g., high macronutrients and current systems) in the different marine environments. For instance, the results of a survey by Ghiglione and colleagues (2012)^58^, which assessed biogeographic patterns in marine microbial communities in polar oceanic waters, support the finding that environmental conditions may influence the structures of microbial communities at different depths. Specifically, the authors found that surface communities may be subject to short-term environmental selection (e.g., temperature and fast-moving surface currents), while deeper communities may be influenced by extended water mass residence, reduced ventilation and sluggish connectivity through ocean circulation^58^. Consequently, the Agulhas Current system’s capacity for driving surface and deep water connectivity^29,37^, may justify the similar trend of F-max and Deep communities. Whereas, the low amounts of dissolved oxygen may be responsible for the observed dissimilarity of the OMZ communities. We note that our decision to pool samples for shotgun analysis based solely on depth may skew the results of community taxonomic and functional analysis based on key marker gene analysis. However, we argue that this decision is unlikely to affect our findings for OMZ and Deep samples as these were from their respective water masses and were likely less variable in comparison to F-max samples.

The differences between bacterial and archaeal communities were ascribed to different depths and stations, a result similar to two distinct Pacific sites^62^. Correlation, variation partitioning and ordination analyses of environmental variables, specifically oxygen (a proxy for respiration), salinity (a proxy for water mass history), depth (a proxy for light penetration) and fluorescence (a proxy for primary productivity) were shown to be important factors for microbial zonation. These results suggest that heterogeneity in these physicochemical properties of the SIO waters may significantly influence the spatial distribution of bacterial and archaeal assemblages. However, we cannot exclude the possibility that other unmeasured biotic and abiotic explanatory variables such as dissolved organic carbon (DOC), particulate organic matter (POM), nitrate, silicate, dimethylsulfoniopropionate (DMSP) and phosphate concentrations may explain the community structure. Alternately, the high proportion of unexplained variability may be due to stochastic dispersal through water masses.

### Microbial communities drive biogeochemical cycling

Microbial primary productivity in the Atlantic and Pacific surface oceans is mainly driven by cyanobacterial lineages, i.e.; *Prochlorococcus* and *Synechococcus*^43,63^. However, the SIO 16S rRNA gene and metagenomic datasets displayed a low relative abundance of reads assigned to *Prochlorococcus* and *Synechococcus*. Despite the low abundance, Cyanobacteria appear to be the primary carbon fixers in surface waters along with Viridiplantae. This finding suggests that, within SIO waters, Cyanobacteria and Viridiplantae may act as keystone species in carbon cycling processes.

Results from metagenome assembly and annotation of the RuBisCO large subunit showed that the majority of ORFs clustered with Form I (closest homology to *Synechococcus* sp. and *Stichococcus* sp.) and IV (Alphaproteobacteria). RuBisCO Form I, II and III are known to catalyse CO_2_ fixation, whereas Form IV gene products have been identified only as RuBisCO-like proteins (RLPs) and lack several amino acid residues involved in catalysis^64^. Further functional characterization of RuBisCOs and RLP proteins may provide insights into how SIO microbial communities use both enzymes to drive photosynthetic and dark carbon fixation. Moreover, our analyses showed that the anaerobic carbon fixation pathway via the reductive TCA cycle may be the dominant energy mechanism in the bacterial communities (mainly Alphaproteobacteria and other lineages; Supplementary Tables S7–S9). The detection of this capacity across all metagenomes suggests a contribution from chemolithoautotrophic metabolism. Potential carbon loss by respiration (indicated by the presence of cytochrome oxidase genes) was also linked to Gammaproteobacteria, Alphaproteobacteria (mostly, unclassified Pelagibacteraceae) and unclassified Planctomycetes (Supplementary Tables S7 and S9).

Nitrogen cycling capacity was dominated by mineralization, assimilation, nitrate reduction and nitrite oxidation pathways, suggesting that microbial communities have the capacity for both organic and inorganic nitrogen utilization. Our analysis showed only low relative abundance levels of nitrogen fixation genes, compared to other genes linked to nitrogen metabolism. *Rhodopseudomonas* sp., a photoorganomixotroph, displayed the metabolic capacity for both denitrification and nitrogen fixation, suggesting that these bacteria may play a role in both nitrogen uptake and release. These results suggest that nitrogen fixation may be driven by non-cyanobacterial members in the SIO, possibly due to iron limitation in this region. Bioavailable iron is a vital co-factor for nitrogen fixation^65^ and a limiting factor for cyanobacterial primary production. The limited iron in the SIO may force other bacterial diazotrophs to fulfil the ecological niche typically occupied by cyanobacteria^66^. The lack of potential nitrifying and ammonia oxidizing bacteria (AOB) in the shotgun metagenomic data supports this observation. However, some archaeal species with a capacity for ammonia oxidation were identified in the 16S rRNA gene Illumina-tagged sequencing and metagenome data. This suggests that these microbial communities may have the capacity for ammonia oxidation, although these taxa may be ecologically rare^67^. We acknowledge that amplification bias may also result in under-representation of these groups^68^.

The analysis of sulfur cycling genes from shotgun analyses showed complete metabolic pathways, assigned to Alpha- and Gammaproteobacteria. Surprisingly, the CR metagenomes yielded no sequences with taxonomic affiliations to the ubiquitous sulfur-oxidizer SUP05 clade, although these phylotypes were observed in the amplicon dataset. The detection of aerobic phototrophic bacterial members of Alphaproteobacteria (*Roseobacter/Rhodobacter* clade), capable of oxidising reduced inorganic sulfur compounds such as sulfite or thiosulfate. In addition, the prevalence of *sox* and other sulfide reductase genes (adenylylsulfate reductase subunit A (*apr*A), adenylylsulfate reductase subunit B (*apr*B) and sulfite reductase (*dsr*A)) in the F-max metagenome further suggests their potential role in phototrophic labile sulfur metabolism^69^.

In summary, our results suggest that free-living microbial assemblages in the SIO are heterogeneous and spatially structured in response to the energetic Agulhas Current and depth-associated physicochemical conditions. Moreover, this oceanographically complex environment is dominated by a few bacterial groups. We identified Gammaproteobacteria as a crucial class responsible for carbon metabolism. Some copiotrophic Gammaproteobacteria are ubiquitous across all depths and have previously been implicated as a sink or source organic matter, and are known to transfer fixed carbon along trophic levels in marine waters^70,71^. These members also appeared to demonstrate a capacity to facilitate the acquisition of inorganic compounds (such as sulfur or nitrate compounds) as an energy source for the fixation of oceanic carbon dioxide (chemolithoautotrophic lifestyle). The dominance of this class in the SIO, a nutrient rich marine environment influenced by Antarctic waters, suggests that they may be uniquely adapted to this region. To a lesser extent, Cyanobacteria and micro-eukaryote (*Viridiplantae*) appeared as principal primary producers. We suggest that the low number of potential autotrophic pathway and nitrogen metabolism genes might be confounded by the paucity of trace metals (especially, iron) and high nutrients in this region^72,73^. Based on the occurrence of RuBisCO and sulfur oxidising genes affiliated to Alphaproteobacteria and Gammaproteobacteria, we predict that these members could play crucial roles in phototrophic productivity. However, there are some caveats associated with the analysis of biogeochemical pathway genes, including the relatively low level of functional annotation from metagenomic reads. Given the relevance of the Agulhas Current system to ocean circulation, further studies may provide clarity on the role of Gammaproteobacteria and viruses in ecosystem functioning and biogeochemistry within the entire ecosystem.

## Materials and Methods

### Sampling site description, sample collection and processing

The Crossroads (CR) transect, established in 2013, was designed to monitor the inter-annual variability of the Agulhas Current and its return path known as the Agulhas Return Current. Activities along the monitoring line include high resolution full depth Conductivity-Temperature-and-Depth (CTD) stations crossing the Agulhas Current system and the Subtropical Front (STF) to its south. Seawater samples were collected during the Marion Island Relief Voyage cruise (15th April − 9th May 2015; austral autumn) on-board the R/V SA. Agulhas II (Fig. 1A). Samples were retrieved from 9 selected oceanographic stations using a 24 × 20 litre Niskin bottle CTD rosette multi-sampler (Sea-Bird SBE-911 plus V2 CTD System; Sea-Bird Electronics, Inc., Bellevue, Washington, USA). Seawater samples (5 l) were immediately collected from 3 Niskin bottles at each station position, from three predetermined depths: (i) Deep (>5 m above seafloor), (ii) Low dissolved oxygen zone (OMZ), and (iii) Fluorescence maximum (F-max). Depth was determined from the altimeter on the CTD rosette and echo-sounders installed aboard the research vessel (Supplementary Table S1). Each 5-litre sample was then pre-filtered through a 47 mm, 0.45-µm-pore cellulose acetate membrane filter to circumvent biomass saturation (Sartorius Stedim Biotech GmbH, Göttingen, Germany), followed by filtration through a 0.2-µm-pore cellulose acetate membrane filter (Sartorius Stedim Biotech, Göttingen, Germany). Temperature, salinity, oxygen and *in situ* fluorescence data was obtained from various sensors contained within the CTD rosette system, for all sampled depths. Filters containing the biomass were stored at −20 °C aboard and transported to CMEG laboratory on ice. In total, we collected 27 samples (n = 27, 9 × 3) for 16S rRNA gene amplicon sequencing, and from these, pooled DNA from each depth (3 in total) for metagenomic analysis.

### Bacterial and Archaeal 16S rRNA gene based amplicon sequencing

Metagenomic DNA was extracted from both filters using a MoBio PowerSoil™ DNA Isolation Kit (MoBio, Carlsbad, CA, USA) according to manufacturer’s instructions. Samples were sent to the Molecular Research DNA (MR DNA) sequencing facility (Shallowater, Texas, USA) for amplicon sequencing of the V4-V5 region with 515 F and 806 R primers on an Illumina MiSeq instrument. This primer pair has been shown to underestimate members of the SAR11 bacterioplankton clade^74^, which could have affected our results. However, our study focused on the variation of microbial communities along depth and in the vicinity of the Agulhas Current system, instead of the resolution of SAR 11 members^37^. Sequence data was analysed using QIIME version 1.9.1^75^. Briefly, fasta and quality files recovered were converted into a single fastq file prior to quality filtering. The metadata mapping file was corrected for any textual or formatting errors. Thereafter, libraries were split into samples using the *split_libraries_fastq.py* script. The detection and removal of chimeric sequences was done using USEARCH v6.1 software^76^, using both the *de novo* and reference-based (Chimera Slayer reference database^77^) methods. The remaining sequences were clustered into OTUs using SILVA 128 database at 97% similarity^78^. Lastly, singletons were filtered out using the *filter_otus_from_otu_table.py* script. Rarefactions curves (Supplementary Fig. S1) were generated in R software (http://cran.r-project.org/; Version 3.2.2). The non-singletons output OTU matrix was normalized to account for uneven sample sums by subsampling all sequences with that of the sample with lowest reads. This rarefaction approach has been shown to favour high abundance taxa and underestimate low abundance/rare taxa^79^. However, our study focuses on the distribution of dominant taxa and their potential role in nutrient cycling, as previously described^37,80^.

### Metagenomic sequencing and Bioinformatic analysis

Metagenomic DNA extracted from each sampled depth was pooled and amplified prior to sequencing on an Illumina HiSeq platform (2 × 250 bp, 20 million reads) at the MR DNA facility (Shallowater, Texas, USA), resulting in a total of 3 samples (n = 3). The quality of reads were assessed and processed using Prinseq-light v0.6^81^: i.e. reads with ambiguous bases and mean phred quality score below 20 were removed from the dataset as previously described^34^. The phi X 174 (ΦX174) bacteriophage^82^ and human reads were removed from the sequencing data using BBMap alignment program (http://sourceforge.net/projects/bbmap). Exact duplicates were also removed to avoid biases resulting from library amplification. The resultant high-quality reads were used for taxonomic and functional analyses. Briefly, paired end reads were joined using PEAR v0.9.10^83^ using default parameters. Thereafter, merged reads were subsampled to the lowest number of reads (for OMZ; 6,605,209) using Seqtk (https://github.com/lh3/seqtk). The resultant reads were searched against the NCBI-NR protein database. Diamond software^84^ was used for BLASTx analysis with an e-value of 1e-3. BLASTx results were then used for taxonomic and functional assignments through the MEGAN v5.10 software^85^. Comparative functional analysis of carbon (C), nitrogen (N) and sulfur (S) metabolism was conducted using KEGG pathway modules and SEED subsystems. Potential microbial C, N and S pathway marker genes (Supplementary Tables S7–S11) were analysed as previously reported^33,34^ with additional RubisCO large subunit phylogenetic analysis^35,86,87^ (Supplementary Fig. S7). Taxonomic classification based on the Small Subunit rRNA gene sequences from the metagenome data were analysed using Metaxa2 profiler^34,88^ and SILVA 128^89^. Viral reads were classified using MEGAN and RefSeq-Virus database^90^.

### Data availability

The raw sequencing reads from this study for the 16S rRNA and shotgun metagenomes have been submitted to the NCBI SRA (accession numbers: SRX2247573: 16S rRNA amplicon data, SRX2185070 (Deep), SRX2185068 (OMZ) and SRX2185067 (F-max) metagenomes.

### Analysis of microbial diversity comparisons

For each sample, alpha diversity and richness indices (Observed OTUs, ACE, Chao1, Shannon and Good’s coverage; Supplementary Table S2) were calculated from rarefied sequences to ensure inter-sample taxonomic comparability using the QIIME *alpha_diversity.py* script. Microbial community analyses using of the 16S rRNA gene, was performed using the *vegan* package^91^ for the statistical program R, to evaluate the relationships between the bacterial/archaeal community structure and environmental parameters (Supplementary Table S1). Beta and gamma diversity calculations were performed using *vegan*. Venn diagrams were generated to illustrate the number of shared and unique OTUs in each sampled depth (F-max, OMZ and Deep) using *vegan* and *gplots* R packages (Supplementary Fig. S2). The ‘core microbiome’, here defined as OTUs (97% OTU cut-off level; Supplementary Fig. S3) which occurred in all samples (F-max1–9, OMZ1–9 and Deep1–9), was obtained using QIIME version 1.9.1^75^. To evaluate the response of microbial communities to the Agulhas Current system, we used relative abundance counts of the sequence data. We note that while this method may be observational and biased in contrast to quantitative data, these methods have provided crucial insights on microbial community structure.

### Statistical analysis

A two-dimensional non-metric multidimensional scaling (NMDS) plot was constructed using Bray-Curtis dissimilarities indices from Hellinger-transformed OTUs (Supplementary Fig. S4), in order to visualize the spatial patterns in community structure among the F-max, OMZ and Deep sample groups, as described previously^91^. Analysis of Similarity (ANOSIM)^92^ was thereafter performed to test for differences between depths based on 999 permutations. The ANOSIM R statistic ranges from 0 to 1, with values closer to 0 indicating more similarity and values closer to 1 indicating more differences. The ANOSIM *p* value was corrected to account for multiple testing according to Benjamini and Hochberg procedure^93^ (Supplementary Table S3). Explanatory variables (i.e., log-transformed environmental variables and depth) and their contribution to Hellinger-transformed community variance partitioning^94^ were determined using the adjusted R^2^ selection criterion with the *varpart* package (Supplementary Figure S5). A redundancy analysis (RDA; Fig. 3) was used to assess the effect of abiotic data using the *ordistep* function using a permutation analysis cut-off *p* value of 0.05, and adjusted R^2^ criteria. Pearson correlations coefficients (r; Supplementary Table S4) and significance tests (*p* < 0.05, Benjamini-Hochberg *p*-correction using the *stats* R package) were used to measure the relationship between environmental variables and microbial communities (at class level; relative abundance, >0.1%). Pearson correlations and heat maps were generated using *Hmisc*, *stats*, *reshape2* and *ggplot2* R packages (Fig. 4). Map representation was performed with Ocean Data View v4.7.6 (Schlitzer, R., Ocean Data View, http://odv.awi.de, 2015).

## Electronic supplementary material

Supplementary Figures
