# Supplementary material for: Agulhas Current properties shape microbial community diversity and potential functionality
Source: Sci Rep. 2018 Jul 12;8:10542. doi: 10.1038/s41598-018-28939-0 (PMC6043601; doi:10.1038/s41598-018-28939-0)
Supplement: Supplementary file 1 — Supplementary Figures [file 41598_2018_28939_MOESM1_ESM.pdf]

Supplementary Information for

Agulhas Current properties shape microbial community diversity and potential functionality

Sandra Phoma[1], Surendra Vikram[1], Janet K. Jansson[2], Isabelle J. Ansorge[3], Don A. Cowan[1], Yves Van de Peer[1,4, 5] and Thulani P. Makhalanyane[1*]

1. Centre for Microbial Ecology and Genomics (CMEG), Department of Biochemistry, Genetics and Microbiology, Natural Sciences 2, University of Pretoria, Pretoria, 0028, South Africa;

2. Earth and Biological Sciences Directorate, Pacific Northwest National Laboratories, P.O. Box 999, Richland, WA, USA;

3. Department of Oceanography and Marine Research Institute (Ma-Re), University of Cape Town, Rondebosch, 7701, South Africa;

4. VIB Centre for Plant Systems Biology, B-9052 Ghent, Belgium;

5. Department of Plant Biotechnology and Bioinformatics, Ghent University, B-9052 Ghent, Belgium;

*Corresponding author: Dr. T.P. Makhalanyane
Centre for Microbial Ecology and Genomics, Department of Genetics, Natural Sciences 2, University of Pretoria, Hatfield, Pretoria, 0028, South Africa
Tel: +2712 420 6976,
Email: thulani.makhalanyane@up.ac.za

Supplementary Figure Legends

Figure S1: Rarefaction curves of operational taxonomic units (OTUs) derived from samples collected along the Crossroads transects. Coloured lines represent each sequenced sample (n=27). The black vertical line depicts the point at which sequence data was subsampled at 34 397 sequence reads. Rarefaction curves were generated at 97% similarity level cut off level in R using the *vegan* package.

Figure S2: Venn diagram displaying shared and exclusive OTU distribution. (A) Oceanic zones and (B) Three water depths. Overlapping areas indicated shared number of OTUs.

Figure S3: Pie-charts indicating the core microbiome present in all CR samples.

Figure S4: Non-metric multidimensional scaling (nMDS) ordination plot showing the reltative similarity of sample groups (F-max, OMZ and Deep) based on community composition (stress = 0.173).

Figure S5: Venn diagrams of the variance partitioning analysis. A model of OTUs including environmental variables (A) and a reduced model using depth and environmental variables (B). Overlapping areas indicate shared variation of the parameter effect on SIO community composition.

Figure S6: Analysis of Small subunit rRNA (SSU) using Metaxa2. Absolute read count of the different phyla is shown on the Y-axis. Taxonomic percent relative abundance of and Illumina shotgun metagenomic reads: (A) Bacterial, (B) Archaeal and (C) Eukaryotic communities.

Figure S7: Maximum likelihood tree of the RubisCO large subunit ORFs recovered from the metagenomic assembly. Green and purple asterisks are showing the type I RusbisCO and Rubisco like proteins (RLPs). The high quality reads were assembled using metaSPAdes v3.7.1 [1]. Contigs longer than 200 bp were selected to predict Open reading framea (ORFs) using Prodigal ORF finder programme in meta mode [2]. Further, the resulting amino acid (aa) sequences were blast searched against custom RuBisCO large subunit proteins [3]. Blastp hits were filtered based on the bitscore (≥100) alignment length longer than 100 aa. Phylogenetic analysis was performed using mafft alignment [4] and Phyml [5]. Resulting maximum likelihood tree was visualized using iTOL [6]. Clades of RubisCO form I, II and III are denoted by green, red and cyan, respectively and RLPs clades are shown in purple.

Figure S1

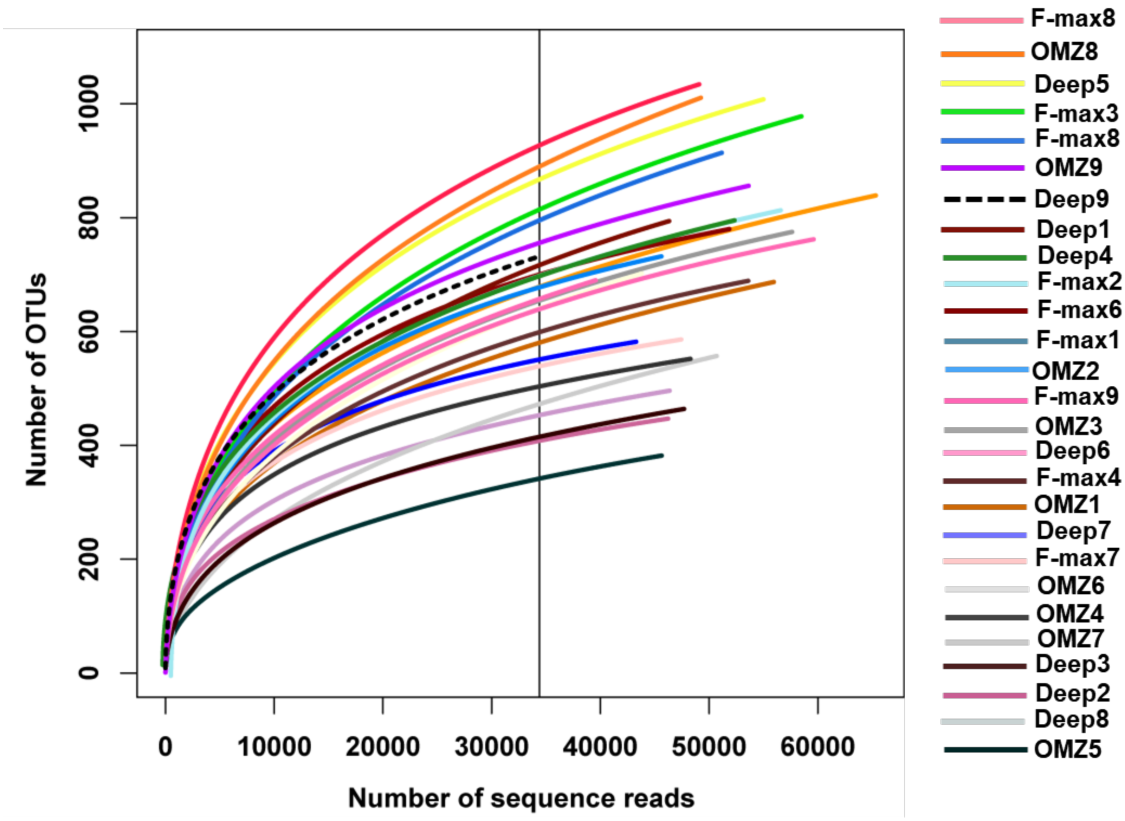

Figure S2

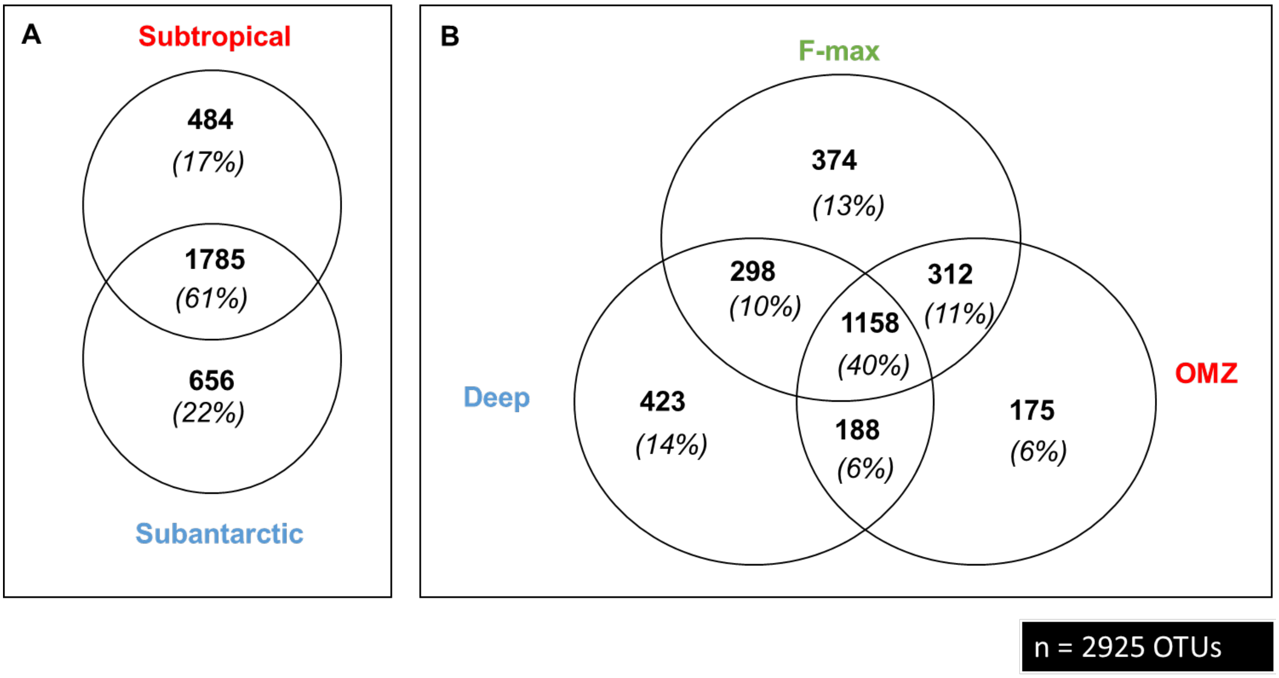

Figure S3

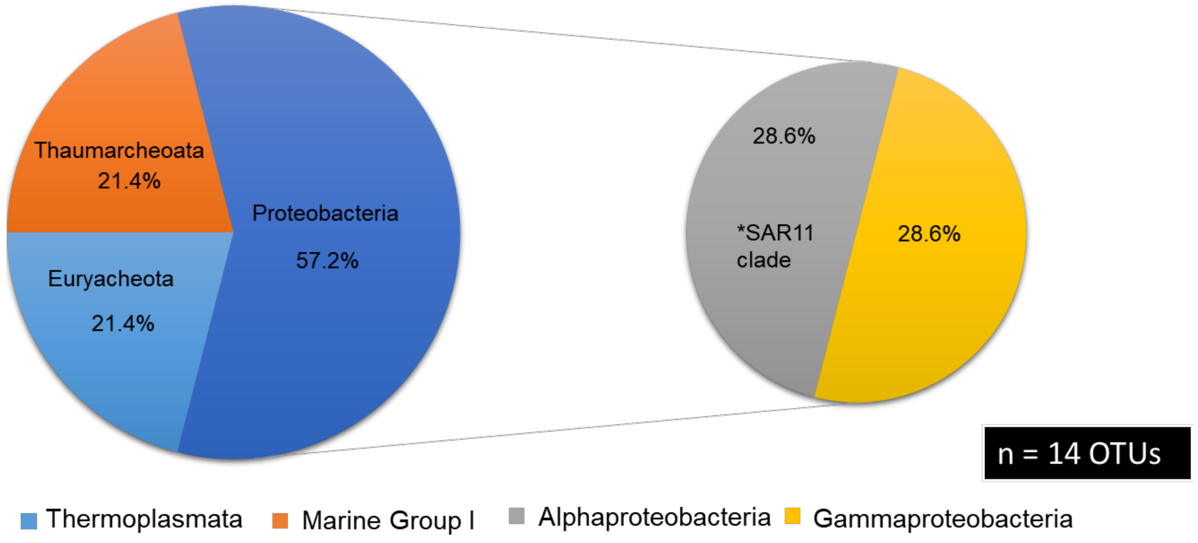

Figure S4

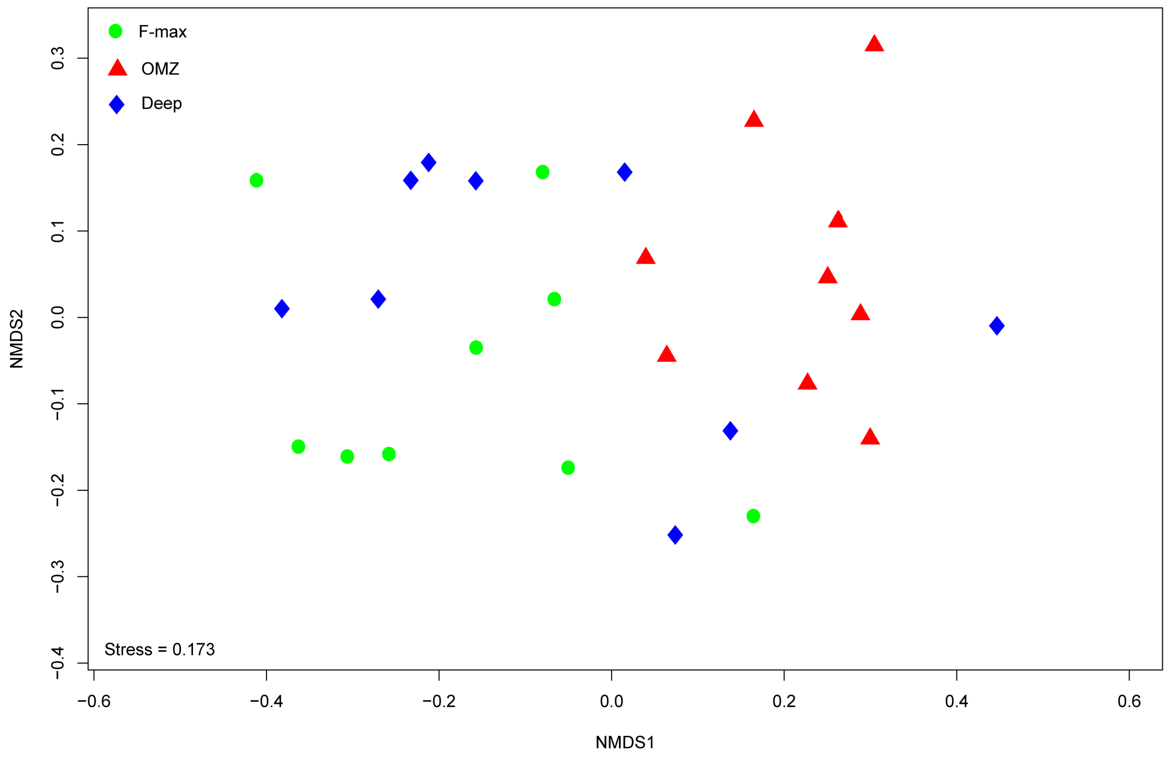

Figure S5

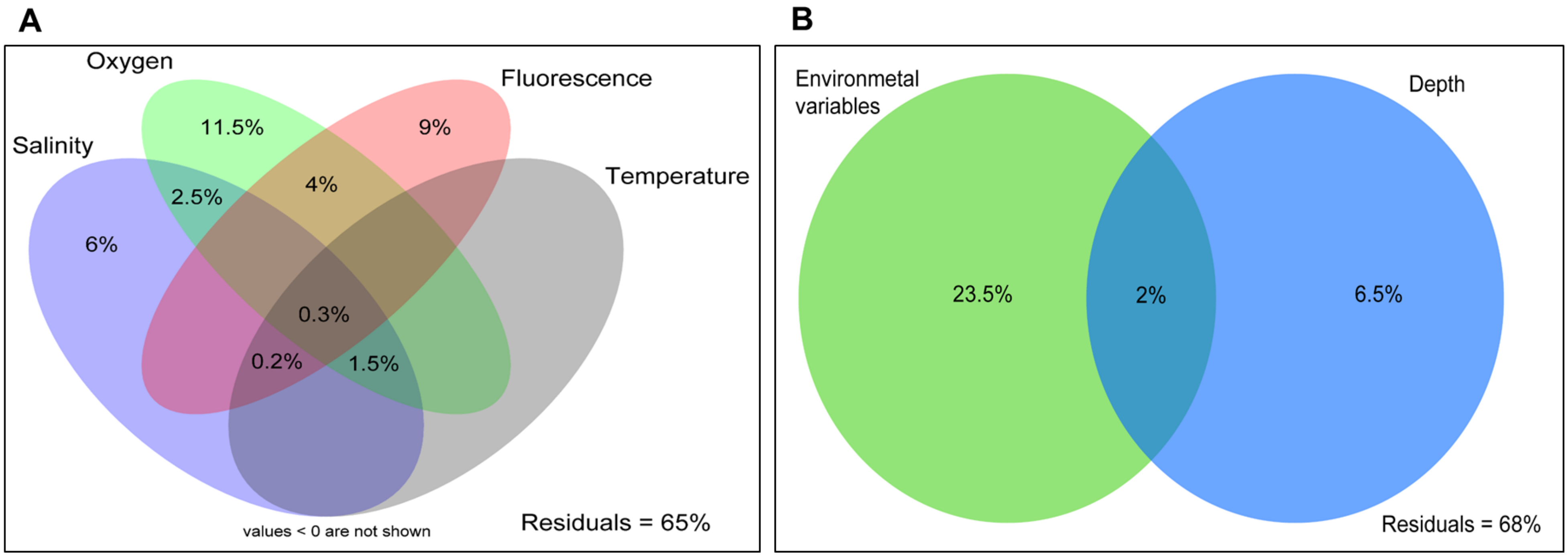
A
Oxygen
Fluorescence
Salinity
Temperature
11.5%
9%
2.5%
4%
6%
0.3%
0.2%
1.5%
values < 0 are not shown
Residuals = 65%
B
Environmetal
variables
Depth
23.5%
2%
6.5%
Residuals = 68%

Figure S6

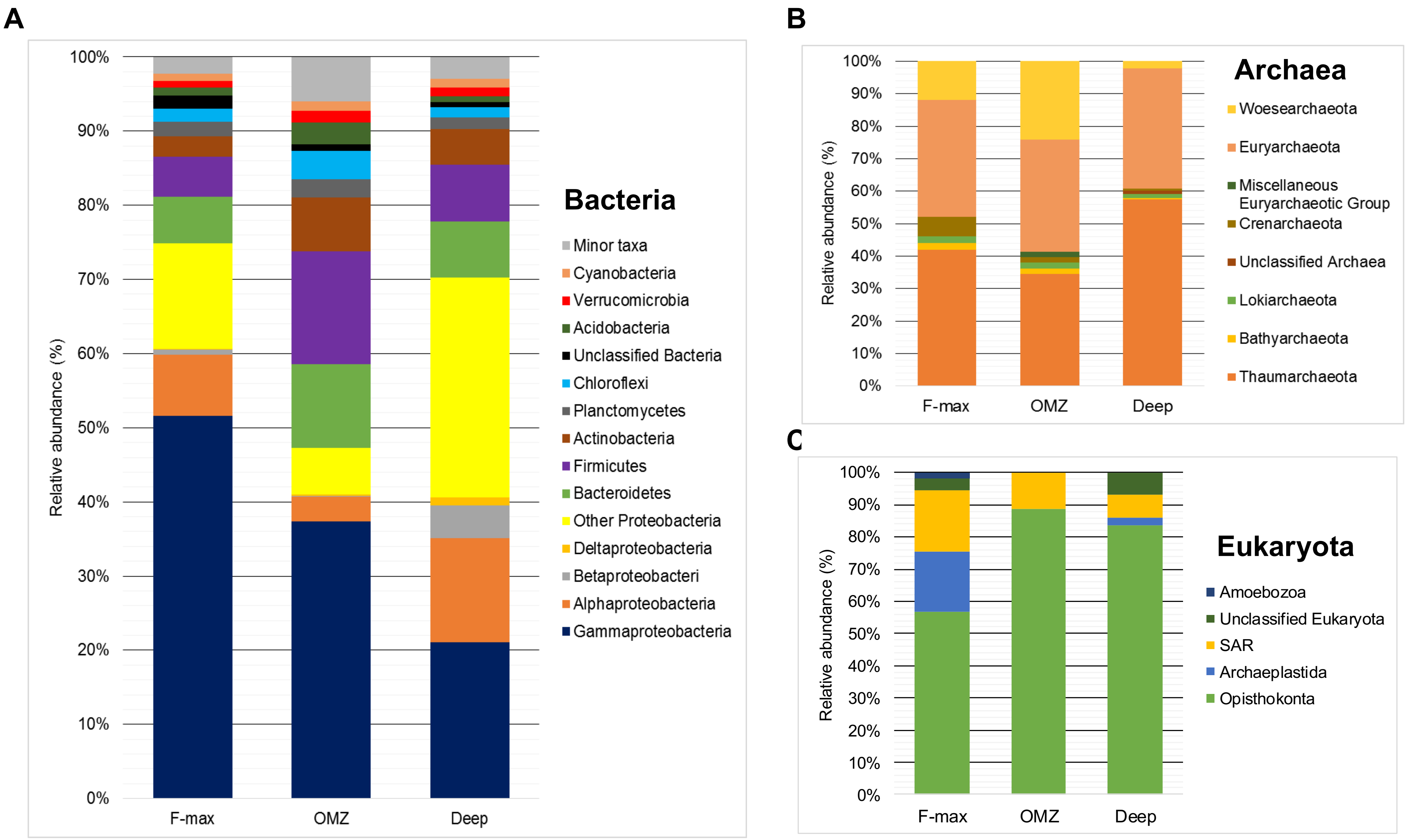
A
Bacteria
Relative abundance (%)
100%
90%
80%
70%
60%
50%
40%
30%
20%
10%
0%
F-max
OMZ
Deep
Minor taxa
Cyanobacteria
Verrucomicrobia
Acidobacteria
Unclassified Bacteria
Chloroflexi
Planctomycetes
Actinobacteria
Firmicutes
Bacteroidetes
Other Proteobacteria
Deltaproteobacteria
Betaproteobacteri
Alphaproteobacteria
Gammaproteobacteria
B
Archaea
Woesearchaeota
Euryarchaeota
Miscellaneous Euryarchaeotic Group
Crenarchaeota
Unclassified Archaea
Lokiarchaeota
Bathyarchaeota
Thaumarchaeota
C
Eukaryota
Amoebozoa
Unclassified Eukaryota
SAR
Archaeplastida
Opisthokonta

Figure S7

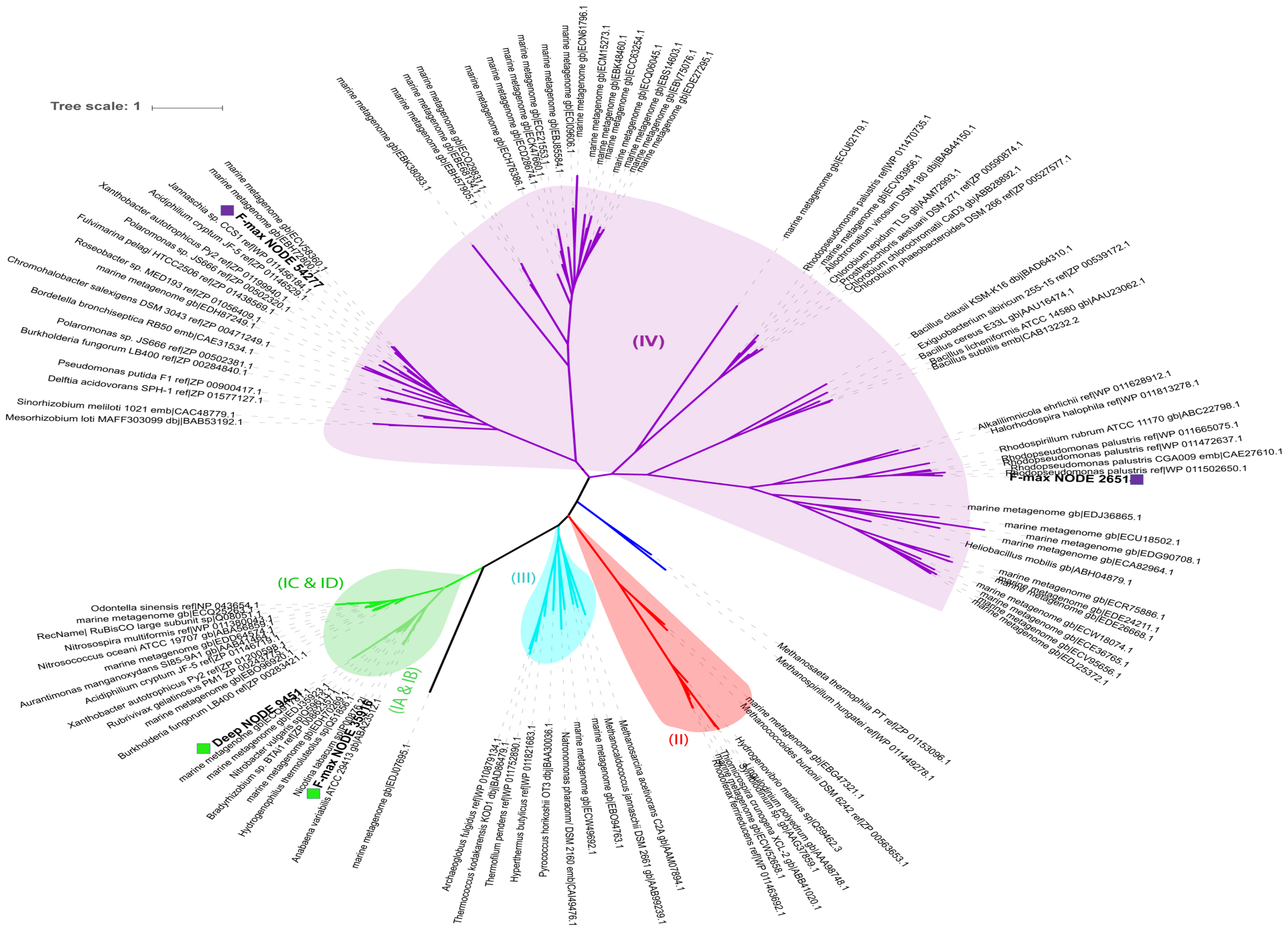

Tree scale: 1
(IV)
(IC & ID)
(IA & IB)
(III)
(II)
F-max NODE 54277
F-max NODE 2651
Deep NODE 9451
F-max NODE 35916
Mesorhizobium loti MAFF303099 dbj|BAB53192.1
Sinorhizobium meliloti 1021 emb|CAC48779.1
Delftia acidovorans SPH-1 ref|ZP 01577127.1
Pseudomonas putida F1 ref|ZP 00900417.1
Heliobacillus mobilis gb|ABH04879.1
marine metagenome gb|EDJ36865.1
Methanosaeta thermophila PT ref|ZP 01153096.1
Methanospirillum hungatei ref|WP 011449278.1

Table S1: Geographical and physicochemical parameters of oceanic samples obtained during the 2015 Marion relief cruise on board the SA Agulhas II R/V

Station number (CR-X)	Sample name	Sample type	Water mass	Sampling date (dd/mm/yy)	Long (E°)	Lat (S°)	Salinity (psu)	Oxygen (ml/l)	Temp (°C)	Fluor (mg/m^3)	Depth (m)	Sounding (m)
CR1	F-max1	F-max	SASW	09/05/15	30.950	-45.999	33.94	6.21	8.66	1.20	24.79	4978.00
	OMZ1	Oxy-min	RSW		30.950	-45.999	34.56	3.73	2.91	0.07	1288.27	
	Deep1	Deep	AABW		30.950	-45.999	34.69	4.67	0.55	0.04	4970.00	
CR2	F-max2	F-max	SASW	10/05/15	29.217	-44.000	34.06	6.10	9.67	1.49	25.79	5361.00
	OMZ2	Oxy-min	RSW		29.217	-44.000	34.54	3.75	2.80	0.05	1282.60	
	Deep2	Deep	AABW		29.217	-44.000	34.66	4.75	0.27	0.02	5350.00	
CR3	F-max3	F-max	STSW	11/05/15	27.625	-42.000	35.19	5.41	14.38	1.29	24.80	3316.00
	OMZ3	Oxy-min	RSW		27.625	-42.000	34.65	3.69	2.97	0.05	1459.25	
	Deep3	Deep	AABW		27.625	-42.000	34.80	4.64	1.92	0.04	3306.00	
CR4	F-max4	F-max	STSW	11/05/15	26.456	-40.500	34.76	5.66	13.24	2.28	34.76	2337.00
	OMZ4	Oxy-min	RSW		26.456	-40.500	34.37	4.66	6.00	0.05	587.59	
	Deep4	Deep	AABW		26.456	-40.500	34.82	4.65	2.31	0.03	2327.60	
CR5	F-max5	F-max	STSW	12/05/15	25.750	-39.500	35.22	5.40	16.43	2.70	34.83	2436.00
	OMZ 5	Oxy-min	RSW		25.750	-39.500	34.53	3.63	3.49	0.05	1119.34	
	Deep5	Deep	AABW		25.750	-39.500	34.82	4.61	2.32	0.03	2428.53	
CR6	F-max6	F-max	STSW	12/05/15	25.043	-38.500	35.43	5.14	17.74	2.35	23.83	3939.00
	OMZ6	Oxy-min	RSW		25.043	-38.500	34.52	3.62	3.49	0.04	1197.41	
	Deep6	Deep	AABW		25.043	-38.500	34.75	4.59	1.36	0.01	3929.35	
CR7	F-max7	F-max	STSW	13/05/15	24.696	-37.998	35.64	4.87	20.02	0.71	24.81	4962.00
	OMZ7	Oxy-min	RSW		24.696	-37.998	34.54	3.99	3.69	0.05	2065.13	
	Deep7	Deep	AABW		24.696	-37.998	34.71	4.57	0.98	0.01	4917.72	
CR8	F-max8	F-max	STSW	13/05/15	23.401	-35.999	35.51	4.69	22.42	0.81	23.82	2074.00
	OMZ8	Oxy-min	RSW		23.401	-35.999	34.60	3.23	4.22	0.05	1642.00	
	Deep8	Deep	AABW		23.401	-35.999	34.73	4.02	2.89	0.04	2064.21	
CR9	F-max9	F-max	STSW	14/05/15	23.075	-35.502	35.40	4.43	22.31	0.63	22.31	1675.00
	OMZ9	Oxy-min	RSW		23.075	-35.502	34.60	3.26	4.08	0.06	1047.64	
	Deep9	Deep	AABW		23.075	-35.502	34.77	4.29	2.69	0.03	1665.89	

CR, Crossroads; SASW, Sub-Antarctic Surface Water; RSW, Red Sea Water; AABW, Antarctic Bottom Water; STSW, Subtropical Surface Water; Long, Longitude; Lat, Latitude; Temp, Temperature; Fluor, Fluorescence.

Table S2: OTU observations, alpha diversity indices and good's coverage at 3% genetic dissimilarity derived from 16S rRNA gene analysis

Station number (CR-X)	Sample name	Sample type	Water mass	High quality reads	OTUs	ACE	Chao-1	PD whole tree	Shannon	Simpson	Good's coverage (%)
**CR1**	F-max1	F-max	SASW	65324	689	1013	1097	3.65	5.06	0.85	99.33
	OMZ1	Oxy-min	RSW	51854	706	897	910	2.34	6.50	0.98	99.44
	Deep1	Deep	AABW	46318	715	1010	940	2.34	5.95	0.95	99.27
**CR2**	F-max2	F-max	SASW	55946	585	873	932	3.63	4.69	0.89	99.40
	OMZ2	Oxy-min	RSW	43284	556	641	642	2.31	5.80	0.95	99.60
	Deep2	Deep	AABW	45610	692	875	858	2.34	4.96	0.87	99.38
**CR3**	F-max3	F-max	STSW	58478	806	1203	1167	2.31	4.94	0.85	99.12
	OMZ3	Oxy-min	RSW	47429	539	702	698	3.75	5.18	0.91	99.57
	Deep3	Deep	AABW	46363	445	602	626	2.34	4.15	0.77	99.57
**CR4**	F-max4	F-max	STSW	50692	486	767	743	2.33	3.91	0.86	99.39
	OMZ4	Oxy-min	RSW	46221	408	569	545	2.34	4.32	0.85	99.62
	Deep4	Deep	AABW	49431	801	1182	1217	2.34	5.17	0.88	99.14
**CR5**	F-max5	F-max	STSW	57635	656	943	906	4.83	5.10	0.89	99.38
	OMZ5	Oxy-min	RSW	45633	343	549	504	3.63	3.63	0.80	99.61
	Deep5	Deep	AABW	58091	867	1248	1219	4.80	6.44	0.97	99.14
**CR6**	F-max6	F-max	STSW	39561	648	998	1065	4.83	6.03	0.96	99.37
	OMZ6	Oxy-min	RSW	48281	491	631	657	2.34	4.47	0.84	99.65
	Deep6	Deep	AABW	55251	701	904	897	3.63	6.41	0.97	99.33
**CR7**	F-max7	F-max	STSW	53593	600	841	827	2.34	5.28	0.92	99.38
	OMZ7	Oxy-min	RSW	47690	418	609	632	2.34	4.29	0.86	99.58
	Deep7	Deep	AABW	52590	686	979	1062	2.31	6.01	0.96	99.34
**CR8**	F-max8	F-max	STSW	49688	968	1288	1306	2.31	6.07	0.94	99.10
	OMZ8	Oxy-min	RSW	49561	942	1322	1358	2.31	6.41	0.96	99.02
	Deep8	Deep	AABW	43805	638	1041	995	2.31	5.55	0.95	99.29
**CR9**	F-max9	F-max	STSW	59610	637	960	948	3.63	4.59	0.81	99.36
	OMZ9	Oxy-min	RSW	53630	772	977	1016	3.63	5.38	0.89	99.30
	Deep9	Deep	AABW	34397	724	933	907	5.08	6.32	0.97	99.40
**Total**	-	-	-	***1355966***	***2925****	-	-		-		-

**A total of 2,925 different OTUs were obtained after all samples were normalised to 34 397 reads; OTUs, operational taxonomic units; PD, Phylogenetic diversity.*

Table S3: Statistical significance of the nMDS plot using ANOSIM analysis

	ANOSIM R	*p*
**All**	0.4544	**0.015**
**F-max vs OMZ**	0.3702	**0.005**
**OMZ vs Deep**	0.2381	**0.005**
**F-max vs Deep**	0.013	0.610

Bold values are significant values ($p < 0.05$); p-values were adjusted using the Benjamini and Hochberg method for multiple testing using the stats R package.

Table S4: Pearson correlation between environmental variables and microbial communities

	Salinity	Oxygen	Temp	Fluorescence	Depth
**Actinobacteria**	0.09	-0.18	0.15	0.34	-0.24*
**Alphaproteobacteria**	0.06	0.02	0.05	-0.13	-0.20***
**Bacteroidetes**	-0.02	-0.25	-0.04	-0.22	-0.29*
**Betaproteobacteria**	-0.01	-0.07	-0.15	-0.14	0.43
**Chloroflexi**	-0.15	0.20	0.11	0.39*	0.01
**Cyanobacteria**	0.22	-0.11	0.12	-0.15	-0.60*
**Deltaproteobacteria**	-0.02	-0.05	-0.15	-0.10	0.47*
**Euryarchaeota**	0.10	-0.11	0.05	0.03	0.18**
**Firmicutes**	-0.34	-0.06	-0.10	-0.06	-0.16*
**Gammaproteobacteria**	-0.08	0.08	-0.08	0.14	-0.38**
**Marinimicrobia (SAR406 clade)**	-0.10	-0.41	-0.08	-0.15	0.06*
**Minor taxa**	0.13	0.22	0.38	0.42*	0.01

*Shaded areas indicate significant levels of Pearson correlation coefficient (r): * = $p < 0.05$; ** = $p < 0.01$ and *** = $p < 0.001$. P-values were adjusted using the Benjamini and Hochberg method for multiple testing using the stats R package. Taxa with insignificant ($p>0.05$) r values were excluded in the heat map (Figure 4).*

	Salinity	Oxygen	Temp	Fluorescence	Depth
**Other Proteobacteria**	0.05	0.01	-0.01	-0.04	-0.28
**Planctomycetes**	0.82**	-0.08	-0.20*	-0.02	-0.74*
**Thaumarchaeota**	-0.13*	0.08	0.08	-0.23*	0.37**
**Unclassified taxa**	-0.03	-0.23	-0.06	0.18	-0.37
**Verrucomicrobia**	0.37	-0.04	0.30	-0.03	-0.15*

*Shaded areas indicate significant levels of Pearson correlation coefficient (r): * = p < 0.05; ** = p < 0.01 and *** = p < 0.001. P-values were adjusted using the Benjamini and Hochberg method for multiple testing using the stats R package. Taxa with insignificant (P>0.05) r values were excluded in the heat map (Figure 4).*

Table S5: Statistics of the raw sequencing data obtained from HiSeq platform

Metagenome	Number of paired end reads	Number of Bases	Average read length
**F-max**	11,039,591 x 2	5.161049429 Gbp	F=234±39; R=233±40
**OMZ**	10,208,473 x 2	4.867812930 Gbp	F=238±33; R=238±33
**Deep**	9,814,116 x 2	4.610049460 Gbp	F=235±39; R=234±40

Table S6: Statistics of the high-quality sequencing reads

Metagenome	Number of Reads merged after PEAR	Number of Bases	Average read length (bp)	Number of reads assigned to KEGG
**F-max**	5,034,761	1.452002494 Gbp	288 ± 97	538,809
**OMZ**	2,672,768	0.683224483 Gbp	255 ± 68	9,340
**Deep**	3,302,119	0.855809175 Gbp	259 ± 82	29,657

Table S7: Presence of Carbon, Nitrogen and Sulfur pathways assigned to taxa in F-max metagenome. The total number of assigned reads is shown in parentheses

Biogeochemical Pathway step	**Gene**	**Taxa**
**Aerobic C fixation**	**Phosphoribulokinase**	***Rhodopseudomonas* sp. (1)**
		Unclassified Gammaproteobacteria (1)
		Unclassified Alteromonadales (5)
		Alteromonas sp. (5)
		Idiomarina sp. (2)
		Pseudoalteromonas sp. (35)
		Gammaproteobacteria bacterium MFB021 (1)
		Synechococcus sp. (16)
		Prochlorococcus (1)
		Unclassified Viridiplantae (3)
	RuBisCO small chain	*Synechococcus* sp. (1)
	RuBisCO Large chain	Unclassified (2)
		Unclassified Bacteria (2)
		Unclassified Proteobacteria (1)
		Unclassified Alphaproteobacteria (1)
		Unclassified Rhizobiales (1)
		Rhodopseudomonas palustris (7)
		Prochlorococcus marinus (1)
		Prochlorococcus sp. (1)
		Unclassified Viridiplantae (3)
		Unclassified Mamiellales (1)
		Unclassified Streptophytina (1)
Anaerobic C fixation	2-oxoglutarate:ferredoxin oxidoreductase subunit alpha	Unclassified Bacteria (24)
		Unclassified Bacteroidetes (4)
		Unclassified Chitinophagaceae (1)
		Unclassified Proteobacteria (1)
		Unclassified Sphingomonadaceae (2)
		Unclassified PVC group (1)
		Unclassified Planctomycetaceae (4)
		Unclassified Rhodopirellula (3)
		Candidatus Actinomarina minuta (8)
		Unclassified Propionibacteriales (1)
	2-oxoglutarate:ferredoxin oxidoreductase subunit beta	Unclassified Thaumarchaeota (1)
		Unclassified Bacteria (3)
		Unclassified Proteobacteria (1)
		Unclassified Alphaproteobacteria (1)
		Unclassified Halieaceae (1)
		Unclassified Planctomycetaceae (3)
		Rhodopirellula sp. (1)
		Candidatus Actinomarina minuta (1)

	frdA; fumarate reductase flavoprotein subunit	Unclassified Proteobacteria (1)
		Unclassified Alphaproteobacteria (7)
		Unclassified Rhizobiales (1)
	adenosinetriphosphate (ATP) citrate lyase	Unclassified Aspergillaceae (1)
		Aspergillus sp. (2)
		Penicillium sp. (4)
		Unclassified Sordariomycetes (1)
		Unclassified Hypocreales (1)
	CO dehydrogenase subunit delta	Nd
	CO dehydrogenase subunit gamma	Nd
Aerobic respiration	cytochrome c oxidase subunit I (coxI)	Unclassified Eukaryota (1)
		Unclassified Viridiplantae (1)
		Unclassfied prasinophytes (6)
	cytochrome c oxidase subunit III (coxIII)	Nd
	cytochrome c oxidase subunit I (coxA)	Unclassified cellular organisms (9)
		Unclassified Thaumarchaeota (1)
		Unclassified Nitrosopumilaceae (1)
		Unclassified Bacteria (23)
		Unclassified Acidobacteria (2)
		Unclassified FCB group (4)
		Unclassified Bacteroidetes (7)
		Unclassified Cytophagales (2)
		Unclassified Flavobacteriia (16)
		Unclasssified Flavobacteriales (12)
		Unclassified Crocinitomicaceae (1)
		Fluviicola taffensis (2)
		Unclassified Flavobacteriaceae (20)
		Flavobacterium sp. (1)
		Unclassified Proteobacteria (14)
		Unclassified Alphaproteobacteria (50)
		Unclassified Pelagibacteraceae (86)
		Candidatus Pelagibacter sp. (6)
		Candidatus Pelagibacter sp. IMCC9063 (19)
		Alpha proteobacterium HIMB5 (1)
		Alpha proteobacterium HIMB59 (5)
		Unclassified Rhizobiales (4)
		Unclassified Bradyrhizobiaceae (3)
		Rhodopseudomonas sp. (2)
		Rhodopseudomonas palustris (1)
		Unclassified Rhizobiaceae (1)
		Rhizobium sp. (2)
		Unclassiifed Rhodobacterales (4)
		Unclassified Rhodobacteraceae (9)

		Paracoccus sp. (1)
		Unclassified Rhodospirillales (1)
		Unclassified Rhodospirillaceae (5)
		Azospirillum sp. (8)
		Spotted fever group (1)
		Unclassified Sphingomonadaceae (1)
		SAR116 cluster (5)
		OM43 clade (1)
		Unclassified Nitrosomonadaceae (1)
		Unclassified Gammaproteobacteria (33)
		Unclassified Alteromonadales (6)
		Unclassified Alteromonadaceae (13)
		Alteromonas sp. (16)
		Alteromonas macleodii (1)
		Pseudoalteromonas sp. (162)
		Pseudoalteromonas haloplanktis (41)
		Pseudoalteromonas lipolytica (38)
		Pseudoalteromonas sp. A2
		Pseudoalteromonas sp. BSi20652 (12)
		Pseudomonas sp. (3)
		Unclassified Planctomycetales (8)
		Unclassified Planctomycetaceae (26)
		Blastopirellula marina (2)
		Pirellula staleyi (5)
		Unclassified Rhodopirellula (11)
		Unclassified Verrucomicrobia (9)
		Unclassified Opitutae (1)
		Coraliomargarita akajimensis (1)
		Coraliomargarita akajimensis DSM 45221 (9)
		Verrucomicrobiae bacterium DG1235 (1)
		Unclassified Terrabacteria group (1)
		Unclassified Actinobacteria (7)
		Prochlorococcus sp. (1)
		Synechococcus sp. (33)
	cytochrome c oxidase subunit III (coxC)	Unclassified Cellular organisms (1)
		Unclassified Bacteria (21)
		Unclassified FCB group (2)
		Unclassified Bacteroidetes (3)
		Flavobacteriia sp. (5)
		Unclassified Flavobacteriales (8)
		Fluviicola taffensis (3)
		Unclassified Flavobacteriaceae (15)
		Salegentibacter sp. Hel I 6 (1)
		Flavobacteria bacterium MS024-2A

		Unclassified Proteobacteria (5)
		Unclassified Alphaproteobacteria (16)
		Unclassified Pelagibacteraceae (59)
		Candidatus Pelagibacter (2)
		Candidatus Pelagibacter sp. IMCC9063 (5)
		Alpha proteobacterium HIMB59 (7)
		Unclassified Rhizobiales (2)
		Unclassified Bradyrhizobiaceae (5)
		Unclassified Rhizobiaceae (1)
		Unclassified Rhodobacteraceae (1)
		Unclassified Rhodospirillaceae (1)
		Azospirillum sp. (1)
		SAR116 cluster (3)
		Unclassified Burkholderiales (1)
		Unclassified Myxococcales (1)
		Unclassified Gammaproteobacteria (5)
		Unclassified Alteromonadales (2)
		Alteromonas sp. (7)
		Idiomarina sp. (1)
		Pseudoalteromonas sp. (145)
		Pseudoalteromonas haloplanktis (3)
		Pseudoalteromonas sp ND6B (2)
		Unclassified Chromatiales (1)
		Pseudomonas aeruginosa (1)
		Unclassified Planctomycetaceae (4)
		Blastopirellula marina DSM 3645 (4)
		Rhodopirellula sp. (3)
		Unclassified Verrucomicrobia (1)
		Unclassified Terrabacteria group (2)
		Unclassified Synechococcales (6)
		Prochlorococcus sp. (1)
		Synechococcus (9)
CO oxidation	CO dehydrogenase small subunit (coxS)	Unclassified cellular organisms (1)
		Unclassified Bacteria (4)
		Candidatus Entotheonella (3)
		Unclassified Proteobacteria (1)
		Unclassified Alphaproteobacteria (4)
		Unclassified Pelagibacteraceae (2)
		Alpha proteobacterium HIMB5 (2)
		Unclassified Bradyrhizobiaceae (1)
		Rhodopseudomonas sp. (1)
		Unclassified Rhodobacteraceae (3)
		Unclassified Betaproteobacteria (1)
		Pirellula staleyi DSM 6068 (1)

		Unclassified Terrabacteria group (1)
		Unclassified Actinobacteria (1)
		Unclassified Propionibacteriales (1)
	cutM, coxM; carbon-monoxide dehydrogenase medium subunit	Unclassified Bacteria (2)
		Unclassified Proteobacteria (2)
		Unclassified Alphaproteobacteria (5)
		Unclassified Pelagibacteraceae (2)
		Alpha proteobacterium HIMB5 (1)
		Unclassified Rhizobiales (1)
		Unclassified Bradyrhizobiaceae (1)
		Bradyrhizobium sp. (2)
		Rhodopseudomonas palustris (2)
		Unclassified Rhodobacteraceae (6)
		Octadecabacter sp. (1)
		SAR324 cluster (1)
	cutL, coxL; carbon-monoxide dehydrogenase large subunit	Unclassified (2)
		Unclassified Bacteria (6)
		Unclassified Proteobacteria (5)
		Unclassified Alphaproteobacteria (11)
		Unclassified Pelagibacteraceae (6)
		Candidatus Pelagibacter ubique (1)
		Alpha proteobacterium HIMB5 (2)
		Unclassified Bradyrhizobiaceae (2)
		Rhodopseudomonas sp. (1)
		Rhodopseudomonas palustris (10)
		Unclassified Rhizobiaceae (2)
		Unclassified Rhodobacterales (1)
		Unclassified Rhodobacteraceae (7)
		Unclassified Rhodospirillales (2)
		Azospirillum sp. (2)
		Unclassified Burkholderiaceae (7)
		Unclassified Comamonadaceae (4)
		Unclassified Actinobacteria (1)
		Unclassified Acidimicrobiales (1)
		Unclassified Actinobacteria (6)
Fermentation	L-lactate dehydrogenase	Unclassified cellular_organisms (1)
		Unclassified PVC group (7)
		Unclassified Planctomycetales (3)
		Unclassified Planctomycetaceae (10)
		Rhodopirellula sp. (6)
		Unclassified Opitutaceae (4)
Nitrogen Fixation	Nitrogenase	*Rhodopseudomonas palustris* (1)
	nitrogenase molybdenum-iron protein alpha chain (nifD)	Unclassified Bacteria (3)

		Unclassified Alphaproteobacteria (1)
		Rhodopseudomonas sp. (1)
		Rhodopseudomonas palustris (2)
	nitrogenase molybdenum-iron protein beta chain (nifK)	Unclassified Alphaproteobacteria (2)
		Rhodopseudomonas palustris (3)
Ammonification	formate-dependent nitrite reductase periplasmic cytochrome c552 (nrfA)	Nd
	cytochrome c nitrite reductase (nrfA)	Nd
Anammox (SRAO)	hydroxylamine oxidoreductase/hydrazine oxidoreducatse (hao/hzo)	Unclassified Nitrosomonadaceae (1)
Denitrification	nitrous oxide reductase (nosZ)	Unclassified Flavobacteriaceae (7)
		Unclassified Bradyrhizobiaceae (2)
		Rhodopseudomonas palustris (1)
	nitric-oxide reductase (norC)	Nd
	nitric-oxide reductase (norB)	*Idiomarina* sp. (2)
		Unclassified Propionibacteriales (2)
Nitrate reduction + Nitrite oxidation	nitrate reductase alpha & nitrite oxidoreductase (narG/nxrA)	Unclassified Gammaproteobacteria (3)
		Unclassified Alteromonadaceae (13)
	nitrate reductase beta & nitrite oxidoreductase (narH/nxrB)	Unclassified Proteobacteria (1)
		Unclassified Gammaproteobacteria (2)
		Unclassified Alteromonadaceae (3)
		Halomonas sp. (6)
Nitrate reduction	periplasmic nitrate reductase (napA)	Nd
	cytochrome c-type protein (napB)	Nd
Nitrification	ammonia monooxygenase subunit A (amoA)	Nd
	ammonia monooxygenase subunit B (amoB)	Nd
	ammonia monooxygenase subunit C (amoC)	Unclassified Thaumarchaeota (1)
Nitrogen assimilation	glutamate synthase (NADPH/NADH) large chain (gltB)	Unclassified (2)
		Unclassified cellular organisms (5)
		Unclassified Bacteria (41)
		Nitrospina sp. (1)
		Unclassified Proteobacteria (18)
		Unclassified Alphaproteobacteria (42)
		Unclassified Pelagibacteraceae (83)
		Candidatus Pelagibacter (1)
		Alpha proteobacterium HIMB59 (25)
		Unclassified Rhizobiales (2)
		Rhodopseudomonas palustris (2)
		Agrobacterium sp. (1)

		Unclassified Rhodobacterales (1)
		Unclassified Rhodobacteraceae (78)
		Planktomarina temperata RCA23 (1)
		Unclassified Rhodospirillales (2)
		Unclassified Acetobacteraceae (2)
		Unclassified Rhodospirillaceae (12)
		Azospirillum sp. (3)
		Unclassified Erythrobacteraceae (1)
		Erythrobacter sp. (1)
		Novosphingobium sp. (1)
		SAR116 cluster (5)
		Candidatus Puniceispirillum marinum (2)
		Burkholderia sp. (1)
		Unclassified Gammaproteobacteria (25)
		Unclassified Alteromonadaceae (1)
		Alteromonas sp. (8)
		Marinobacter sp. (1)
		Idiomarina sp. (9)
		Pseudoalteromonas sp. (111)
		Unclassified Chromatiales (1)
		Unclassified Chromatiaceae (1)
		Thioalkalivibrio sp. (1)
		Unclassified Enterobacterales (1)
		Unclassified Erwiniaceae (1)
		Unclassified Morganellaceae (1)
		Unclassified Oceanospirillales (1)
		Alcanivorax sp. (7)
		Unclassified Moraxellaceae (6)
		Psychrobacter sp. (284)
		Psychrobacter arcticus (6)
		Psychrobacter cryohalolentis (6)
		Psychrobacter sp. PRwf-1 (1)
		Pseudomonas sp. (2)
		Unclassified Planctomycetaceae (34)
		Rhodopirellula sp. (16)
		Unclassified Terrabacteria group (2)
		Unclassified Actinobacteria (Class) (3)
		Candidatus Actinomarina minuta (9)
		Unclassified Corynebacteriales (4)
	glutamate synthase (ferredoxin-dependent) (gltS)	Unclassified cellular organisms (4)
		Unclassified Bacteria (25)
		Unclassified Flavobacteriia (36)
		Unclassified Flavobacteriaceae (35)
		Cellulophaga sp. (2)

		Flavobacterium sp. (2)
		Mesonia mobilis (1)
		Salegentibacter sp. Hel I 6 (5)
		Flavobacteria bacterium MS024-2A (10)
		Coraliomargarita akajimensis (20)
		Unclassified Terrabacteria group (3)
		Unclassified Cyanobacteria (1)
		Unclassified Oscillatoriales (1)
		Prochlorococcus sp. (1)
		Prochlorococcus marinus (2)
		Synechococcus sp. (41)
		Unclassified Viridiplantae (1)
		Unclassified Chlorophyta (2)
		Unclassified Mamiellophyceae (1)
		Unclassified Trebouxiophyceae (1)
	assimilatory nitrate reductase (K00360)	Nd
	Assimilatory nitrate reductase (K00367)	Unclassified Bacteria (4)
		Unclassified Bacteroidetes (1)
		Unclassified Flavobacteriaceae (5)
	Glutamine synthetase (glnA)	Unclassified (15)
		Unclassified cellular organisms (7)
		Unclassified Thaumarchaeota (4)
		Unclassified Bacteria (57)
		Unclassified Bacteroidetes (8)
		Bacteroides sp. (1)
		Unclassified Cytophagales (2)
		Unclassified Cytophagaceae (4)
		Unclassified Flavobacteriia (4)
		Unclassified Crocinitomicaceae (2)
		Unclassified Flavobacteriaceae (32)
		Flavobacterium sp. (1)
		Unclassified Proteobacteria (21)
		Unclassified Alphaproteobacteria (22)
		Unclassified Pelagibacteraceae (83)
		Candidatus Pelagibacter (1)
		Candidatus Pelagibacter sp. IMCC9063 (4)
		Candidatus Pelagibacter ubique (8)
		Alpha proteobacterium HIMB5 (1)
		Alpha proteobacterium HIMB59 (24)
		Unclassified Rhizobiales (6)
		Unclassified Bradyrhizobiaceae (1)
		Bradyrhizobium sp. (1)
		Mesorhizobium sp. (1)

		Unclassified Rhizobium/Agrobacterium group (1)
		Unclassified Rhodobiaceae (1)
		Unclassified Rhodobacteraceae (40)
		Paracoccus sp. (2)
		Planktomarina temperate sp. (1)
		Planktomarina temperata RCA23 (7)
		Unclassified Rhodospirillales (1)
		Unclassified Acetobacteraceae (1)
		Unclassified Rhodospirillaceae (3)
		Unclassified Sphingomonadales (1)
		Unclassified Betaproteobacteria (3)
		Burkholderia sp. (1)
		Unclassified Nitrosomonadaceae (2)
		Unclassified Gammaproteobacteria (9)
		Unclassified Alteromonadaceae (1)
		Alteromonas sp. (8)
		Marinobacter sp. (2)
		Pseudoalteromonas sp. (16)
		Pseudoalteromonas haloplanktis (1)
		Pseudoalteromonas lipolytica (1)
		Unclassified Cellvibrionales (2)
		Unclassified Halieaceae (1)
		Thioalkalivibrio (1)
		Unclassified Oceanospirillales (2)
		Unclassified Halomonadaceae (3)
		Halomonas sp. (1)
		Halomonas meridian (2)
		Unclassified Oceanospirillaceae (2)
		Psychrobacter sp. (38)
		Psychrobacter arcticus (1)
		Unclassified Pseudomonadaceae (3)
		Pseudomonas sp. (11)
		Pseudomonas stutzeri subgroup (1)
		Candidatus Thioglobus singularis (2)
		Unclassified Vibrionaceae (1)
		Unclassified Rhodanobacteraceae (1)
		Unclassified Planctomycetales (3)
		Singulisphaera acidiphila (2)
		Unclassified Planctomycetaceae (6)
		Unclassified Rhodopirellula (2)
		Unclassified Verrucomicrobia (2)
		Coraliomargarita akajimensis (1)
		Unclassified Spirochaetia (1)
		Unclassified Terrabacteria group (2)

		Unclassified Actinobacteria (Class) (6)
		Candidatus Actinomarina minuta (2)
		Unclassified Cyanobacteria (2)
		Unclassified Synechococcales (1)
		Prochlorococcus sp. (1)
		Synechococcus sp. (17)
		Unclassified Bacilli (1)
		Unclassified Eukaryota (6)
		Penicillium sp. (2)
		Unclassified Sordariomycetes (1)
		Unclassified Oomycetes (1)
		Unclassified Mamiellales (2)
		Unclassified Embryophyta (1)
		Unclassified rosids (1)
Nitrogen Mineralization	glutamate dehydrogenase (K00260)	Unclassified cellular organisms (1)
		Unclassified Bacteria (1)
		Verrucomicrobia bacterium SCGC AAA164-L15 (1)
		Unclassified Firmicutes (1)
	glutamate dehydrogenase (K00261)	Unclassified cellular organisms (5)
		Unclassified Bacteria (6)
		Uncultured marine bacterium EB0 49D07 (1)
		Unclassified Cytophagales (1)
		Unclassified Flavobacteriaceae (1)
		Unclassified Proteobacteria (1)
		Unclassified Alphaproteobacteria (3)
		Candidatus Pelagibacter ubique (2)
		Unclassified Rhizobiaceae (2)
		Unclassified Rhodobacteraceae (10)
		Leisingera sp (1)
		Unclassified Ectothiorhodospiraceae (1)
		Unclassified Planctomycetales (1)
		Unclassified Planctomycetaceae (23)
		Pirellula staleyi (2)
		Rhodopirellula sp. (10)
		Unclassified Terrabacteria group (1)
	glutamate dehydrogenase (K00262)	Unclassified Bacteria (10)
		Unclassified FCB group (1)
		Unclassified Bacteroidetes (2)
		Unclassified Flavobacteriia (2)
		Unclassified Flavobacteriales (5)
		Unclassified Flavobacteriaceae (27)
		Unclassified Sphingomonadales (1)
		Unclassified Gammaproteobacteria (3)

		Unclassified Alteromonadales (1)
		Unclassified Alteromonadaceae (1)
		Alteromonas sp. (6)
		Alteromonas macleodii (3)
		Alteromonas mediterranea (1)
		Alteromonas mediterranea 615 (3)
		Marinobacter sp. (2)
		Idiomarina sp. (5)
		Pseudoalteromonas sp. (4)
		Pseudoalteromonas atlantica (4)
		Unclassified Moraxellaceae (1)
		Psychrobacter sp. (76)
		Psychrobacter arcticus (25)
		Psychrobacter cryohalolentisi (4)
		Psychrobacter sp. G (3)
		Unclassified Piscirickettsiaceae (1)
		Unclassified Cyanobacteria (1)
Assimilatory sulfate reduction	adenylylsulfate kinase (cysC)	Unclassified root (7)
		Unclassified cellular organisms (3)
		Unclassified Bacteria (17)
		Unclassified FCB group (1)
		Salegentibacter sp. Hel I 6 (6)
		Unclassified Pelagibacteraceae (8)
		Candidatus Pelagibacter ubique (1)
		Unclassified Gammaproteobacteria (4)
		Alteromonas sp. (2)
		Pseudoalteromonas sp. (14)
		Pseudoalteromonas sp. SCSIO 11900 (11)
		Unclassified Chromatiaceae (1)
		Alcanivorax sp. (1)
		Unclassified Verrucomicrobia (4)
		Coraliomargarita akajimensis (1)
		Mycobacterium sp. (1)
		Synechococcus sp. (7)
		Unclassified Eurotiomycetidae (1)
		Penicillium sp. (2)
		Unclassified Chlorophyta (1)
	sulfate adenylyltransferase subunit 1 (cysN)	Unclassified Bacteria (6)
		Unclassified Bacteroidetes (8)
		Unclassified Cytophagales (2)
		Unclassified Flavobacteriaceae (4)
		Unclassified Leeuwenhoekiella (1)
		Salegentibacter sp. Hel I 6 (1)
		Unclassified Proteobacteria (2)

		Unclassified Betaproteobacteria (1)
		Methylophilales bacterium HTCC2181 (1)
		Unclassified Epsilonproteobacteria (1)
		Unclassified Gammaproteobacteria (4)
		Unclassified Alteromonadales (2)
		Alteromonas sp. (4)
		Pseudoalteromonas sp. (40)
		Unclassified Chromatiaceae (1)
		Unclassified Halomonadaceae (1)
		Unclassified Verrucomicrobia (4)
		Unclassified Opitutae (1)
	sulfate adenylyltransferase subunit 2 (cysD)	Unclassified Bacteria (13)
		Unclassified Bacteroidetes (1)
		Unclassified Flavobacteriaceae (3)
		Unclassified Proteobacteria (8)
		Unclassified Alphaproteobacteria (2)
		Alpha proteobacterium HIMB59 (3)
		Unclassified Rhizobiales (1)
		Rhodopseudomonas sp. (1)
		Unclassified Hyphomicrobiaceae (1)
		Unclassified Betaproteobacteria (1)
		Unclassified Alcaligenaceae (1)
		Unclassified Gammaproteobacteria (7)
		Unclassified Alteromonadales (27)
		Marinobacter sp. (1)
		Pseudoalteromonas sp. (34)
		Pseudoalteromonas lipolyticai (2)
		Pseudoalteromonas sp. BSi20429 (2)
		Unclassified Halomonadaceae (2)
		Halomonas sp. (3)
		Unclassified Rhodanobacteraceae (1)
		Rhodopirellula sp. (5)
		Unclassified Verrucomicrobia (1)
		Coraliomargarita akajimensis (2)
Dissimilatory sulfate reduction and sulfide oxidation	adenylylsulfate reductase subunit A (aprA)	Unclassified (3)
		Unclassified Proteobacteria (1)
		Unclassified Pelagibacteraceae (70)
	adenylylsulfate reductase subunit B (aprB)	Unclassified (3)
		Unclassified Pelagibacteraceae (39)
	sulfite reductase (dsrA)	Unclassified Bacteria (1)
Sulfur Mineralization	cysteine dioxygenase	Unclassified Cellular organisms (1)
		Penicillium sp. (1)
		dsDNA viruses, no RNA stage (1)
	3-mercaptopyruvate sulfurtransferase	Unclassified cellular organisms (2)

		Unclassified Thaumarchaeota (1)
		Unclassified Bacteria (16)
		Unclassified Proteobacteria (7)
		Unclassified Alphaproteobacteria (3)
		Unclassified Pelagibacteraceae (4)
		Candidatus Pelagibacter (3)
		Candidatus Pelagibacter ubique (4)
		Alpha proteobacterium HIMB5 (1)
		Rhodopseudomonas palustris (1)
		Unclassified Rhodobacteraceae (11)
		Unclassified Rhodospirillales (1)
		SAR116 cluster (1)
		Candidatus Puniceispirillum marinum (1)
		SAR116 cluster alpha proteobacterium HIMB100 (1)
		Unclassified Betaproteobacteria (1)
		Unclassified Gammaproteobacteria (4)
		Alteromonas sp. (7)
		Pseudoalteromonas sp. (19)
		Marine gamma proteobacterium HTCC2148 (1)
		Marine gamma proteobacterium HTCC2143 (2)
		Pseudomonas sp. (1)
		Unclassified Actinobacteria (Class) (1)
Polysulfide reduction	polysulfide reductase chain A (psrA)	Unclassified cellular organisms (1)
		Unclassified Bacteria (1)
		Unclassified Verrucomicrobia (1)
		Unclassified Terrabacteria group (1)
		Unclassified Actinobacteria (1)

Nd = Not detected

Table S8: Presence of Carbon, Nitrogen and Sulfur pathways assigned to taxa in OMZ metagenome. Number of reads assigned are written in parentheses

Biogeochemical Pathway step	Gene	Taxa
Aerobic C fixation	Phosphoribulokinase	*Rhodopseudomonas* sp. (5)
	RuBisCO small chain	Unclassified Gammaproteobacteria (1)
	RuBisCO Large chain	*Rhodopseudomonas* sp. (2)
		Rhodopseudomonas palustris (1)
		Unclassified Gammaproteobacteria (1)
Anaerobic C fixation	2-oxoglutarate:ferredoxin oxidoreductase subunit alpha	Unclassified Bacteria (1)
		Candidatus Actinomarina minuta (2)

	2-oxoglutarate:ferredoxin oxidoreductase subunit beta	Unclassified Bacteria (1)
		Unclassified Bacteroidetes (1)
	frdA; fumarate reductase flavoprotein subunit	Nd
	adenosinetriphosphate (ATP) citrate lyase	Unclassified Eurotiomycetidae (1)
		Unclassified Aspergillaceae (1)
		Penicillium sp. (2)
	CO dehydrogenase subunit delta	Nd
	CO dehydrogenase subunit gamma	Nd
Aerobic respiration	cytochrome c oxidase subunit I (coxI)	Nd
	cytochrome c oxidase subunit III (coxIII)	Nd
	cytochrome c oxidase subunit I (coxA)	Unclassified Thaumarchaeota (1)
		Unclassified Cytophagales (1)
		Unclassified Flavobacteriales (1)
		Unclassified Alphaproteobacteria (2)
		Unclassified Rhizobiales (1)
		Unclassified Bradyrhizobiaceae (5)
		Rhodopseudomonas sp. (1)
		Unclassified Gammaproteobacteria (1)
	cytochrome c oxidase subunit III (coxC)	Unclassified Alphaproteobacteria (1)
		Unclassified Bradyrhizobiaceae (4)
		Unclassified Betaproteobacteria (1)
		Unclassified Terrabacteria group (1)
CO oxidation	CO dehydrogenase small subunit (coxS)	Unclassified Rhizobiales (1)
		Unclassified Bradyrhizobiaceae (2)
		Rhodopseudomonas sp. (2)
		SAR324 cluster (1)
	cutM, coxM; carbon-monoxide dehydrogenase medium subunit	Unclassified Bradyrhizobiaceae (1)
		Rhodopseudomonas palustris (3)
	cutL, coxL; carbon-monoxide dehydrogenase large subunit	Unclassified Bacteroidetes (1)
		Unclassified Bradyrhizobiaceae (6)
		Bradyrhizobium sp. (1)
		Rhodopseudomonas palustris (5)
		Rhodopseudomonas palustris HaA2 (11)
Fermentation	L-lactate dehydrogenase	Nd
Nitrogen Fixation	Nitrogenase	Nd
	nitrogenase molybdenum-iron protein alpha chain (nifD)	Unclassified Bacteria (1)
		Unclassified Alphaproteobacteria (1)
		Rhodopseudomonas sp. (1)
	nitrogenase iron protein (nifH)	Unclassified Alphaproteobacteria (2)
		Unclassified Rhizobiales (1)
		Rhodopseudomonas sp. (1)

		Rhodopseudomonas palustris (1)
	nitrogenase molybdenum-iron protein beta chain (nifK)	*Rhodopseudomonas* sp. (3)
		Rhodopseudomonas palustris (1)
		Rhodopseudomonas palustris BisB5 (1)
Ammonification	formate-dependent nitrite reductase periplasmic cytochrome c552 (nrfA)	Nd
	cytochrome c nitrite reductase (nrfA)	Nd
Anammox (SRAO)	hydroxylamine oxidoreductase/hydrazine oxidoreducatse (hao/hzo)	Nd
Denitrification	nitrous oxide reductase (nosZ)	Unclassified Bacteria (3)
		Unclassified Rhizobiales (1)
		Unclassified Bradyrhizobiaceae (3)
		Rhodopseudomonas palustris (14)
	nitric-oxide reductase (norC)	Nd
	nitric-oxide reductase (norB)	Nd
Nitrate reduction + Nitrite oxidation	nitrate reductase alpha & nitrite oxidoreductase (narG/nxrA)	Unclassified Actinobacteria (1)
		Microbacterium trichothecenolyticum (3)
	nitrate reductase beta & nitrite oxidoreductase (narH/nxrB)	Nd
Nitrate reduction	periplasmic nitrate reductase (napA)	Unclassified Enterobacteriaceae (1)
	cytochrome c-type protein (napB)	*Escherichia coli* (1)
Nitrification	ammonia monooxygenase subunit A (amoA)	Nd
	ammonia monooxygenase subunit B (amoB)	Nd
	ammonia monooxygenase subunit C (amoC)	Nd
Nitrogen assimilation	glutamate synthase (NADPH/NADH) large chain (gltB)	Alpha proteobacterium HIMB59 (1)
		Unclassified Bradyrhizobiaceae (1)
		Rhodopseudomonas sp. (6)
		Rhodopseudomonas palustris (3)
	glutamate synthase (ferredoxin-dependent) (gltS)	Unclassified Flavobacteriia (1)
		Coraliomargarita akajimensis (1)
		Unclassified Terrabacteria group (1)
	assimilatory nitrate reductase	Nd
	glutamine synthetase (glnA)	Unclassified (1)
		Cellular organisms (1)
		Unclassified Bacteroidetes (1)
		Unclassified Alphaproteobacteria (2)
		Unclassified Pelagibacteraceae (1)
		Unclassified Bradyrhizobiaceae (3)
		Rhodopseudomonas sp. (4)
		Rhodopseudomonas palustris (3)

		Rhodopseudomonas palustris HaA2 (1)
		Rhodopirellula sp. (1)
		Unclassified Actinobacteria (Class) (1)
		Microbacterium sp. (1)
Nitrogen Mineralization	glutamate dehydrogenase (K00260)	Nd
	glutamate dehydrogenase (K00261)	Unclassified Actinobacteria (Class) (1)
	glutamate dehydrogenase (K00262)	Unclassified Flavobacteriaceae (1)
Assimilatory sulfate reduction	adenylylsulfate kinase (cysC)	Nd
	sulfate adenylyltransferase subunit 1 (cysN)	Unclassified Bacteroidetes (1)
	sulfate adenylyltransferase subunit 2 (cysD)	Unclassified Alphaproteobacteria (1)
Dissimilatory sulfate reduction and sulfide oxidation	adenylylsulfate reductase subunit A (aprA)	Unclassified Pelagibacteraceae (2)
	adenylylsulfate reductase subunit B (aprB)	Unclassified Pelagibacteraceae (1)
	sulfite reductase (dsrA)	Nd
Sulfur Mineralization	cysteine dioxygenase	Nd
	3-mercaptopyruvate sulfurtransferase	Unclassified Bacteria (2)
		Unclassified Alphaproteobacteria (1)
		Unclassified Bradyrhizobiaceae (1)
		Rhodopseudomonas sp. (2)
		Unclassified Gammaproteobacteria (1)
		Unclassified Spongiibacteraceae (1)
Polysulfide reduction	polysulfide reductase chain A (psrA)	Nd
Nitrogen assimilation	glutamate synthase (NADPH/NADH) large chain (*glt*B)	Alpha proteobacterium HIMB59 (1)
		Unclassified Bradyrhizobiaceae (1)
		Rhodopseudomonas sp. (6)
		Rhodopseudomonas palustris (3)
	glutamate synthase (ferredoxin-dependent) (*glt*S)	Unclassified Flavobacteriia (1)
		Coraliomargarita akajimensis (1)
		Unclassified Clostridiales (1)
	assimilatory nitrate reductase	Nd
	glutamine synthetase (*gln*A)	Unclassified (1)
		Cellular organisms (1)
		Unclassified Bacteroidetes (1)
		Unclassified Alphaproteobacteria (2)
		Unclassified Pelagibacteraceae (1)
		Unclassified Bradyrhizobiaceae (1)
		Rhodopseudomonas sp. (5)
		Rhodopseudomonas palustris (3)
		Rhodopseudomonas palustris HaA2 (1)
		Rhodopirellula sp. (1)
		Unclassified Actinobacteria class (1)
		Microbacterium sp. (1)

Nitrogen Mineralization	glutamate dehydrogenase (K00260)	Nd
	glutamate dehydrogenase (K00261)	Unclassified Actinobacteria class (1)
	glutamate dehydrogenase (K00262)	Unclassified Flavobacteriaceae (1)
Assimilatory sulfate reduction	adenylylsulfate kinase (*cys*C)	Nd
	sulfate adenylyltransferase subunit 1 (*cys*N)	Unclassified Bacteroidetes (1)
	sulfate adenylyltransferase subunit 2 (*cys*D)	Unclassified Alphaproteobacteria (1)
Dissimilatory sulfate reduction and sulfide oxidation	adenylylsulfate reductase subunit A (*apr*A)	Unclassified Pelagibacteraceae (2)
	adenylylsulfate reductase subunit B (*apr*B)	Unclassified Pelagibacteraceae (1)
	sulfite reductase (*dsr*A)	Nd
Sulfur Mineralization	cysteine dioxygenase	Nd
	3-mercaptopyruvate sulfurtransferase	Unclassified Bacteria (2)
		Unclassified Alphaproteobacteria (1)
		Unclassified Bradyrhizobiaceae (1)
		Rhodopseudomonas sp. (2)
		Unclassified Gammaproteobacteria (1)
		Unclassified Spongiibacteraceae (1)
Polysulfide reduction	polysulfide reductase chain A (*psr*A)	Nd

Nd = Not detected

Table S9: Presence of Carbon, Nitrogen and Sulfur pathways assigned to taxa in Deep metagenome. Number of reads assigned are written in parentheses.

Biogeochemical Pathway step	Gene	Taxa
Aerobic C fixation	Phosphoribulokinase	Unclassified Synechococcales (1)
	RuBisCO small chain	Unclassified Synechococcaceae (1)
		Synechococcus sp. (1)
	RuBisCO Large chain	Unclassified Cellular organisms (1)
		Unclassified Bacteria (1)
		Synechococcus sp. (2)
		Prochlorococcus sp. (1)
Anaerobic C fixation	2-oxoglutarate:ferredoxin oxidoreductase subunit alpha	Unclassified Bacteria (6)
		Unclassified Chitinophagaceae (1)
		Unclassified Hyphomonadaceae (2)
		Unclassified Planctomycetaceae (2)
		Rhodopirellula sp. (1)
		Candidatus Actinomarina minuta (3)
	2-oxoglutarate:ferredoxin oxidoreductase subunit beta	Unclassified Bacteria (2)
		Unclassified Bacteroidetes/Chlorobi group (2)
		Unclassified Chitinophagaceae (2)

		Unclassified Rhodocyclaceae (1)
		Unclassified Planctomycetaceae (1)
		Pirellula staleyi (1)
	frdA; fumarate reductase flavoprotein subunit	Nd
	adenosinetriphosphate (ATP) citrate lyase	Nd
	CO dehydrogenase subunit delta	Nd
	CO dehydrogenase subunit gamma	Nd
Aerobic respiration	cytochrome c oxidase subunit I (coxI)	Nd
	cytochrome c oxidase subunit III (coxIII)	Unclassified Viridiplantae (1)
		Ostreococcus sp. (1)
	cytochrome c oxidase subunit I (coxA)	Unclassified Thaumarchaeota (1)
		Unclassified Bacteria (1)
		Unclassified Bacteroidetes (1)
		Unclassified Flavobacteriales (2)
		Unclassified Flavobacteriaceae (4)
		Gemmatirosa kalamazoonesis (1)
		Unclassified Proteobacteria (1)
		Unclassified Pelagibacteraceae (6)
		Candidatus Pelagibacter sp. IMCC9063 (1)
		Alpha proteobacterium HIMB59 (1)
		Unclassified Rhizobiales (1)
		Methylobacterium sp. (1)
		Rhodobacteraceae (1)
		Unclassified Chromobacteriaceae (1)
		Unclassified Bdellovibrionales (2)
		Unclassified Gammaproteobacteria (1)
		Unclassified Planctomycetaceae (3)
		Rhodopirellula sp. (2)
		Unclassified Verrucomicrobia (4)
		Verrucomicrobia bacterium SCGC AAA168-F10 (1)
		Terrabacteria group (1)
		Synechococcus sp. (7)
	cytochrome c oxidase subunit III (coxC)	Unclassified Bacteria (5)
		Unclassified Cytophagales (1)
		Unclassified Flavobacteriia (3)
		Unclassified Flavobacteriaceae (1)
		Arenibacter algicola (1)
		Flavobacteria bacterium MS024-2A (1)
		Uncultured Marinimicrobia bacterium HF0010 18O13 (1)
		Unclassified Pelagibacteraceae (4)
		Alpha proteobacterium HIMB59 (1)
		Limnobacter sp. MED105 (1)

		Bacteriovorax sp. BAL6 X (1)
		Unclassified Gammaproteobacteria (1)
		Unclassified Planctomycetaceae (1)
		Rubinisphaera brasiliensis (1)
		Unclassified Verrucomicrobia (1)
		Unclassified Chloroflexi (1)
		Synechococcus sp. (2)
CO oxidation	CO dehydrogenase small subunit (coxS)	Unclassified Proteobacteria (1)
		Unclassified Rhodospirillales (1)
	cutM, coxM; carbon-monoxide dehydrogenase medium subunit	*Methylobacterium* sp. (1)
	cutL, coxL; carbon-monoxide dehydrogenase large subunit	Unclassified Alphaproteobacteria (1)
		Unclassified Pelagibacteraceae (1)
Fermentation	L-lactate dehydrogenase	Unclassified Bacteria (2)
		PVC group (1)
		Unclassified Planctomycetales (1)
		Unclassified Planctomycetaceae (1)
		Unclassified Opitutaceae (3)
Nitrogen Fixation	Nitrogenase	Nd
	nitrogenase molybdenum-iron protein alpha chain (nifD)	Nd
	nitrogenase iron protein (nifH)	Nd
	nitrogenase molybdenum-iron protein beta chain (nifK)	Nd
Ammonification	formate-dependent nitrite reductase periplasmic cytochrome c552 (nrfA)	Nd
	cytochrome c nitrite reductase (nrfA)	Nd
Anammox (SRAO)	hydroxylamine oxidoreductase/hydrazine oxidoreducatse (hao/hzo)	Nd
Denitrification	nitrous oxide reductase (nosZ)	Nd
	nitric-oxide reductase (norC)	Nd
	nitric-oxide reductase (norB)	Nd
Nitrate reduction + Nitrite oxidation	nitrate reductase alpha & nitrite oxidoreductase (narG/nxrA)	Unclassified Alphaproteobacteria (5)
		Erythrobacter longus (5)
	nitrate reductase beta & nitrite oxidoreductase (narH/nxrB)	Unclassified Gammaproteobacteria (1)
Nitrate reduction	periplasmic nitrate reductase (napA)	Nd
	cytochrome c-type protein (napB)	Nd
Nitrification	ammonia monooxygenase subunit A (amoA)	Nd
	ammonia monooxygenase subunit B (amoB)	Unclassified Betaproteobacteria (1)

	ammonia monooxygenase subunit C (amoC)	Nd
Nitrogen assimilation	glutamate synthase (NADPH/NADH) large chain (gltB)	Unclassified Bacteria (4)
		Unclassified Bacteroidetes (1)
		Unclassified Chitinophagaceae (5)
		Unclassified Proteobacteria (1)
		Unclassified Alphaproteobacteria (3)
		Unclassified Pelagibacteraceae (7)
		Candidatus Pelagibacter ubique (2)
		Alpha proteobacterium HIMB59 (2)
		Unclassified Rhodobacterales (1)
		Unclassified Rhodobacteraceae (5)
		Unclassified Rhodospirillales (2)
		Unclassified Burkholderiales (1)
		Unclassified Burkholderiaceae (1)
		Limnobacter sp. MED105 (2)
		Unclassified Comamonadaceae (1)
		Unclassified Gammaproteobacteria (2)
		Psychrobacter sp. (1)
		Pseudomonas pelagia (1)
		Unclassified Planctomycetaceae (3)
		Rhodopirellula sp. (2)
		Unclassified Actinobacteria (Class) (1)
		Candidatus Actinomarina minuta (1)
	glutamate synthase (ferredoxin-dependent) (gltS)	Unclassified Cellular organisms (1)
		Unclassified Bacteria (4)
		Unclassified Flavobacteriia (5)
		Unclassified Flavobacteriaceae (5)
		Flavobacterium sp. (1)
		Mesonia mobilis (1)
		Flavobacteria bacterium MS024-2A (2)
		Coraliomargarita akajimensisi (1)
		Unclassified Rhodobacteraceae (1)
		Candidatus Actinomarina minuta (1)
		Unclassified Chroococcales (1)
		Synechococcus sp. (3)
		Unclassified Viridiplantae (1)
		Unclassified Chlorophyceae (1)
	assimilatory nitrate reductase	Nd
	glutamine synthetase (glnA)	Unclassified (1)
		Unclassified Thaumarchaeota (2)
		Nitrosopumilus sp. (1)
		Unclassified Bacteria (8)
		Unclassified Chitinophagaceae (1)

		Unclassified Flavobacteriia (1)
		Unclassified Flavobacteriaceae (3)
		Unclassified Proteobacteria (2)
		Unclassified Alphaproteobacteria (4)
		Unclassified Pelagibacteraceae (8)
		Candidatus Pelagibacter sp. IMCC9063 (1)
		Candidatus Pelagibacter ubique (1)
		Alpha proteobacterium HIMB59 (2)
		Unclassified Rhodobacteraceae (2)
		Unclassified Sphingomonadales (2)
		SAR116 cluster (1)
		Desulfovibrio sp. (1)
		Pseudomonas sp. (3)
		Methylophaga sp. (2)
		Unclassified Planctomycetales (1)
		Singulisphaera acidiphila (1)
		Unclassified Actinobacteria (Class) (1)
		Candidatus Actinomarina minuta (1)
		Synechococcus sp. (3)
		Unclassified Deinococci (1)
Nitrogen Mineralization	glutamate dehydrogenase (K00260)	Nd
	glutamate dehydrogenase (K00261)	Unclassified Cellular organisms (2)
		Unclassified Bacteria (4)
		Unclassified Cytophagaceae (1)
		Unclassified Cytophagales (1)
		Unclassified Alphaproteobacteria (2)
		Unclassified Bradyrhizobiaceae (1)
		Afipia sp. (3)
		Unclassified Planctomycetaceae (3)
		Rhodopirellula sp. (2)
	glutamate dehydrogenase (K00262)	Unclassified Bacteria (1)
		Unclassified Bacteroidetes (2)
		Unclassified Flavobacteriales (1)
		Methylophaga nitratireducenticrescens (1)
		Unclassified Terrabacteria group (1)
		Unclassified Propionibacteriaceae (1)
		Penicillium sp. (1)
Assimilatory sulfate reduction	adenylylsulfate kinase (cysC)	Unclassified Thaumarchaeota (1)
		Unclassified Bacteria (3)
		Bacteroidetes/Chlorobi_group (2)
		Unclassified Proteobacteria (1)
		Unclassified Pelagibacteraceae (1)
	sulfate adenylyltransferase subunit 1 (cysN)	Unclassified Bacteria (1)
		Escherichia coli (1)

		Unclassified Verrucomicrobia (2)
		Coraliomargarita akajimensis (1)
	sulfate adenylyltransferase subunit 2 (cysD)	Unclassified Bacteria (2)
		Rhodopirellula sp. (1)
Dissimilatory sulfate reduction and sulfide oxidation	adenylylsulfate reductase subunit A (aprA)	Unclassified (1)
		Unclassified Pelagibacteraceae (8)
	adenylylsulfate reductase subunit B (aprB)	Unclassified Pelagibacteraceae (2)
	sulfite reductase (dsrA)	Nd
Sulfur Mineralization	cysteine dioxygenase	Nd
	3-mercaptopyruvate sulfurtransferase	Unclassified Cellular organisms (1)
		Unclassified Nitrosopumilaceae (1)
		Unclassified Bacteria (1)
		Unclassified Proteobacteria (1)
		Unclassified Alphaproteobacteria (1)
		Unclassified Pelagibacteraceae (3)
		Unclassified Rhizobiales (1)
		Marine gamma proteobacterium HTCC2143 (1)
		Hyphomicrobium sp. (1)
		Alcanivorax sp. (2)
		Pseudomonas pelagia (1)
Polysulfide reduction	polysulfide reductase chain A (psrA)	Unclassified Bacteria (1)

Nd = Not detected

Table S10: Genes for the Photosystem-I and II from three different depths (F-max, OMZ and Deep) metagenomes. The number of assigned reads is shown in parentheses.

**Photosystem-I**			
**Gene**	**F-max**	**OMZ**	**Deep**
psaA	Unclassified Cellular organisms (1)	Nd	Unclassified Cellular organisms (1)
	Prochlorococcus (1)		
	Synechococcus sp. (43)		*Synechococcus* sp. (1)
	Unclassified Chlamydomonadales (1)		
psaB	Unclassified Synechococcales (1)	Nd	*Synechococcus* sp. (1)
	Synechococcus sp. (11)		
	Unclassified Eukaryota (1)		
	Unclassified Viridiplantae (1)		
psaC	Unclassified Cellular organisms (1)	Nd	Nd
	Unclassified Cyanobacteria (5)		
	Prochlorococcus sp. (1)		
	Synechococcus sp. (2)		
	Unclassified Viridiplantae (2)		

psaD	*Synechococcus* sp. (7)	Nd	Nd
	Unclassified Mesangiospermae (2)		
psaE	Unclassified Synechococcales (1)	Nd	*Synechococcus* sp. (1)
	Unclassified Synechococcaceae (1)		
	Unclassified Viridiplantae (1)		
	Unclassified Chlorophyta (2)		
psaF	*Synechococcus* sp. (6)	Nd	Nd
psaG	Nd	Nd	Nd
psaI	*Synechococcus* sp. (1)	Nd	Nd
psaJ	*Synechococcus* sp. (2)	Nd	Nd
psaK	*Synechococcus* sp. (4)	Nd	Nd
psaL	*Synechococcus* sp. (10)	Nd	Nd
	Unclassified Chlorophyta (1)		
psaM	*Synechococcus* sp. (1)	Nd	*Synechococcus* sp. (1)
psaN	Unclassified Bathycoccaceae (1)	Nd	Nd
psaO	Unclassified Chlorophyta (1)	Nd	Nd
**Photosystem-II**			
**Gene**	**F-max**	**OMZ**	**Deep**
psbA	Unclassified (38)	Unclassified (1)	Unclassified (8)
	Unclassified Cyanobacteria (2)		Unclassified Cyanobacteria (2)
	Unclassified Nostocales (1)		*Prochlorococcus* (1)
	Unclassified Microcoleaceae (1)		*Prochlorococcus marinus* (1)
	Unclassified Synechococcaceae (1)		*Synechococcus* sp. (27)
	Unclassified Viruses (4)		
	Unclassified T4-like viruses (2)		
	Unclassified Myoviridae (2)		
	Synechococcus sp. (32)		
psbB	Unclassified Cellular organisms (2)	Nd	Nd
	Unclassified Prochlorococcus (1)		
	Unclassified Synechococcaceae (1)		
	Synechococcus sp. (5)		
	Unclassified Eukaryota (1)		
	Unclassified Viridiplantae (1)		
psbC	*Synechococcus* sp. (7)	Nd	Unclassified Synechococcales (1)
	Unclassified Chlorophyta (2)		
psbD	Unclassified (6)	Nd	*Synechococcus* sp. (2)
	Unclassified Cellular organisms (7)		
	Unclassified Synechococcales (2)		
	Prochlorococcus sp. (2)		
	Unclassified Synechococcaceae (1)		
	Synechococcus sp. (24)		
	Synechococcus sp. CC9902 (4)		

	Synechococcus sp. KORDI-49 (1)		
	Unclassified Eukaryota (1)		
	Unclassified Viruses (2)		
	Unclassified Caudovirales (4)		
	Unclassified Myoviridae (4)		
	Unclassified T4-like viruses (1)		
	Unclassified Myoviridae (1)		
	Unclassified Siphoviridae (1)		
psbE	*Synechococcus* sp. (5)	Nd	Nd
psbF	*Prochlorococcus* sp. (1)	Nd	Nd
	Synechococcus sp. (1)		
psbH	*Synechococcus* sp. (3)	Nd	Nd
psbJ	Cellular organisms (4)	Nd	Nd
psbK	*Synechococcus* sp. CC9902 (1)	Nd	Nd
psbL	Unclassified Chlorophyta (1)	Nd	Nd
psbM	*Prochlorococcus* sp. (1)		
psbO	Unclassified Bacteria (4)	Nd	Nd
	Prochlorococcus sp. (1)		
	Synechococcus sp. (2)		
	Unclassified Chlorophyta (1)		
psbP	*Synechococcus* sp. (9)	Nd	*Synechococcus* sp. (1)
	Unclassified Eukaryota (1)		Unclassified Viridiplantae (1)
	Unclassified Viridiplantae (4)		Unalssified Embryophyta (1)
	Unclassified Chlorophyta (3)		
	Unclassified Chlamydomonadales (1)		
	Unclassified Mamiellales (1)		
	Micromonas sp. (1)		
psbR	*Viridiplantae* sp. (1)	Nd	Nd
psbS	Unclassified Chlorophyta (1)	Nd	Unclassified Chlorophyta (2)
psbU	*Synechococcus* (4)	Nd	Nd
psbV	*Synechococcus* (1)	Nd	*Synechococcus* sp. (1)
psbY	*Synechococcus* sp. (5)	Nd	*Synechococcus* sp. (1)
	Unclassified Viridiplantae (1)		
psbZ	*Synechococcus* (1)	Nd	Nd
psb27	*Synechococcus* sp. (1)	Nd	Nd
	Micromonas sp. (1)		
psb28	Unclassified Cellular organisms (2)	Nd	*Synechococcus* sp. (1)
	Synechococcus sp. (6)		
	Unclassified Viridiplantae (2)		

Nd = Not detected

Table S11: Genes for the sox system. Number of reads assigned are written in parentheses.

Gene	CRS	CRM	CRD
soxA	Bacteroidetes/Chlorobi group (1)	Nd	Unclassified Halieaceae (1)
	Unclassified Alphaproteobacteria (1)		
	Bradyrhizobium sp. (1)		
	Unclassified Rhodobacterales (3)		
	Unclassified Hyphomonadaceae (1)		
	Unclassified Rhodobacteraceae (3)		
	Unclassified Gammaproteobacteria (2)		
	Unclassified Halieaceae (1)		
soxB	Unclassified Bacteria (8)	SAR324 cluster (1)	Nd
	Unclassified Rhodobacterales (1)		
	Unclassified Rhodobacteraceae (15)		
	Unclassified Burkholderiaceae (1)		
soxD	Unclassified Proteobacteria (1)	Nd	Unclassified *Rhodobacteraceae* (1)
	Unclassified Alphaproteobacteria (2)		
	Unclassified Rhodobacterales (1)		*Limnobacter* sp. MED105 (1)
	Unclassified Rhodobacteraceae (9)		
	Unclassified Gammaproteobacteria (1)		
soxH	Unclassified Rhodobacterales (1)	Nd	Unclassified Rhodobacteraceae (1)
	Unclassified Rhodobacteraceae (4)		
soxS	Nd	Nd	Unclassified Rhodobacteraceae (1)
			Unclassified Roseovarius (1)
soxR	Unclassified Alphaproteobacteria (2)	Nd	Nd
	Unclassified Rhodobacteraceae (3)		
soxW	Unclassified Rhodobacteraceae (2)	Nd	Unclassified Rhodobacteraceae (1)
	Unclassified Betaproteobacteria (1)		*Limnobacter* sp. MED105 (1)
	Unclassified Gammaproteobacteria (1)		
soxX	Unclassified Bacteria (1)	Nd	Unclassified Rhizobiales (3)
	Unclassified Proteobacteria (1)		
	Unclassified Rhodobacteraceae (4)		
	Rhodobacteraceae bacterium HIMB11 (2)		
	Limnobacter sp. MED105 (1)		
	Unclassified Halieaceae (1)		
soxY	Unclassified Rhodobacteraceae (2)	Nd	Nd
	Unclassified Betaproteobacteria (1)		
	Unclassified Halieaceae (1)		
soxZ	Unclassified Bacteria (2)	Nd	Nd
	Unclassified Rhodobacteaceae (7)		
	Unclassifed Halieaceae (1)		

Nd = Not detected

References:

1 Nurk, S., Meleshko, D., Korobeynikov, A. & Pevzner, P. metaSPAdes: a new versatile de novo metagenomics assembler. *arXiv preprint arXiv:1604.03071* (2016).

2 Hyatt, D. *et al.* Prodigal: prokaryotic gene recognition and translation initiation site identification. *BMC bioinformatics* **11**, 1 (2010).

3 Tabita, F. R., Hanson, T. E., Satagopan, S., Witte, B. H. & Kreel, N. E. Phylogenetic and evolutionary relationships of RubisCO and the RubisCO-like proteins and the functional lessons provided by diverse molecular forms. *Philosophical transactions of the Royal Society of London. Series B, Biological sciences* **363**, 2629-2640, doi:10.1098/rstb.2008.0023 (2008).

4 Katoh, K. & Standley, D. M. MAFFT multiple sequence alignment software version 7: improvements in performance and usability. *Molecular biology and evolution* **30**, 772-780, doi:10.1093/molbev/mst010 (2013).

5 Guindon, S. *et al.* New algorithms and methods to estimate maximum-likelihood phylogenies: assessing the performance of PhyML 3.0. *Systematic biology* **59**, 307-321 (2010).

6 Letunic, I. & Bork, P. Interactive Tree Of Life (iTOL): an online tool for phylogenetic tree display and annotation. *Bioinformatics* **23**, 127-128 (2007).
